# Loci and natural alleles for cadmium-mediated growth responses revealed by a genome wide association study and transcriptome analysis in rice

**DOI:** 10.1186/s12870-021-03145-9

**Published:** 2021-08-13

**Authors:** Jianping Yu, Chaolei Liu, Hai Lin, Bin Zhang, Xiaoxia Li, Qiaoling Yuan, Tianjiao Liu, Huiying He, Zhaoran Wei, Shilin Ding, Chao Zhang, Hongsheng Gao, Longbiao Guo, Quan Wang, Qian Qian, Lianguang Shang

**Affiliations:** 1 Shenzhen Branch, Guangdong Laboratory of Lingnan Modern Agriculture, Genome Analysis Laboratory of the Ministry of Agriculture and Rural Affairs, Agricultural Genomics Institute at Shenzhen, Chinese Academy of Agricultural Sciences, Shenzhen, China; 2 Key Laboratory of Crop Heterosis and Utilization, Ministry of Education/ Beijing Key Laboratory of Crop Genetic Improvement, China Agricultural University, Beijing, 100193 China; 3 State Key Laboratory of Rice Biology, China National Rice Research Institute, Chinese Academy of Agricultural Sciences, Hangzhou, 310006 China

**Keywords:** Cadmium-mediated growth responses, Genome-wide association study, Natural haplotypes, Transcriptome analysis, Glutathione S-transferase

## Abstract

**Background:**

Cadmium (Cd) is a toxic heavy metal that is harmful to the environment and human health. Cd pollution threatens the cultivation of rice (*Oryza sativa* L.) in many countries. Improving rice performance under Cd stress could potentially improve rice productivity.

**Results:**

In this study, 9 growth traits of 188 different cultivated rice accessions under normal and Cd stress conditions were found to be highly variable during the seedling stage. Based on ~3.3 million single nucleotide polymorphisms (SNPs), 119 Cd-mediated growth response (CGR) quantitative trait loci (QTL) were identified by a genome-wide association study (GWAS), 55 of which have been validated by previously reported QTL and 64 were new CGR loci. Combined with the data from the GWAS, transcriptome analysis, gene annotations from the gene ontology (GO) Slim database, and annotations and functions of homologous genes, 148 CGR candidate genes were obtained. Additionally, several reported genes have been found to play certain roles in CGRs. Seven Cd-related cloned genes were found among the CGR genes. Natural elite haplotypes/alleles in these genes that increased Cd tolerance were identified by a haplotype analysis of a diverse mini core collection. More importantly, this study was the first to uncover the natural variations of 5 GST genes that play important roles in CGRs.

**Conclusion:**

The exploration of Cd-resistant rice germplasm resources and the identification of elite natural variations related to Cd-resistance will help improve the tolerance of current major rice varieties to Cd, as well as provide raw materials and new genes for breeding Cd-resistant varieties.

**Supplementary Information:**

The online version contains supplementary material available at 10.1186/s12870-021-03145-9.

## Background

Cadmium (Cd) is a well-known heavy metal element that has a long decomposition cycle, easy migration, high toxicity, and is difficult to degrade, thereby posing a major threat to environmental safety and harming human health through the food chain in the form of biological enrichment [1]. Cd results in harmful pathogenic, carcinogenic, and mutagenesis effects on the human body, including bone pain caused by chronic Cd poisoning [2, 3]. Cd is not an essential element for plant growth and has adverse effects on growth. When Cd accumulates to a certain extent, plants exhibit toxic symptoms, stunted root growth, and inhibited absorption of water and nutrients, leading to a series of physiological metabolic disorders, including blocked chlorophyll, sugar and protein synthesis, decreased photosynthesis, and altered enzymatic activities, which ultimately lead to reduced yield [4]. Cd destroys plant roots by altering RNA synthesis and proton pump activity, and can replace essential elements, including sulfur, calcium, and magnesium, thereby leading to a shortage of these essential elements [5]. Cd mainly binds to proteins in plants and affects their growth and development by interfering with enzymatic activities, resulting in growth disorders [6]. However, the genetic basis of Cd effects on rice growth remains poorly studied.

Recently, several rice genes have been reported to be involved in the uptake and transport of Cd, including *OsIRT1* [7], *OsIRT2*, *OsNRAMP1 *[8], *OsNramp5 *[9], *OsHMA3* [10], *OsHMA2* [11], *OsLCT1* [12], *CAL1* [13], *OsCd1* [14], *OsZIP1 *[15], *LCD* [16], and *OsCCX2* [17]. Among them, *OsNramp5* are responsible for transporting Cd from the apoplast to root cells. *OsHMA3* in the tonoplast selectively sequestrates Cd into vacuoles and *OsHMA2* and *CAL1* transport Zn and Cd from the roots to shoots. *OsLCT1*, a transporter gene for Cd transport in the phloem, regulates grain Cd accumulation. However, to date, only 3 quantitative trait loci (QTL) (i.e., *OsHMA3*, *CAL1*, and *OsCd1*) have been cloned [10, 13, 14]. Three genes (i.e., *OsPCS1*, *OsPCS2*, and *OsCLT1)* have been found to be involved in the chelation of Cd [18–20] and OsPCS2 was more strongly activated by Cd than by As(III) [21–23]. Additionally, the auxin transporter, OsAUX1, is involved in primary root and root hair elongation and Cd stress responses in rice [24]. Although our knowledge of the genetic control of Cd uptake and accumulation in rice has gradually increased, the molecular characteristics of these genes and other genes remain to be identified.

Phytoremediation techniques for Cd contamination in soil has received increasing attention [25, 26]. It has been reported that different rice varieties have different Cd tolerance [27, 28]. In order to cultivate Cd-tolerant rice varieties, it is important to explore the genetic factors that control Cd-mediated growth responses (CGRs). Genome-wide association study (GWAS) is an effective tool for exploiting elite alleles that control important agronomic traits in germplasm resources [29–32]. Wu et al. [33] performed a GWAS using 100 barley accessions and identified 9 QTL for root Cd, 21 for shoot Cd, 15 for grain Cd, and 14 for root-to-shoot Cd translocation. Zhao et al. [34] detected 14 QTL for Cd accumulation in rice grain from a collection of 312 rice accessions by a GWAS. Recently, the grain Cd accumulation QTL, *OsCd1*, which belongs to the major facilitator superfamily, was identified by a GWAS based on 127 rice cultivars [14]. Thus, GWAS for CGRs would aid us to identify more excellent natural variations related to Cd-tolerant.

This study investigated the responses of rice growth to high Cd stress. Based on a GWAS and transcriptome analysis, Cd-tolerant germplasm resources were explored and elite natural variations associated with Cd-resistance were identified. Collectively, these results serve as a resource for potentially improving the tolerance of current major cultivars to Cd and provide useful information for cloning candidate genes in future studies.

## Results

### Phenotypic variation of 9 seedling growth traits under normal and Cd stress conditions

A collection of 188 cultivated rice accessions, consisting of 80 *indica*, 83 temperate *japonica*, and 25 tropical *japonica*, obtained from a mini-core collection of rice in China and other regions of the world, were used in this study [35, 36] (Table S1). This collection represents a large variation of geographical origins and genetic diversity. Various root and shoot traits at the seedling stage were investigated under normal and Cd stress conditions (Table S1), including the sum of root length (SRL), root area (RA), superficial area of roots (SA), root volume (RV), root diameter (RD), maximum root length (MRL), shoot length (SL), shoot weight (SW), and root weight (RW). Compared to normal conditions, 8 traits (i.e., SRL, RA, SA, RV, MRL, SL, SW, and RW) decreased in varying degrees after Cd treatment (Table 1), while 5 traits (i.e., SRL, RA, SA, RV, and RW) were more sensitive, especially RV. These results were consistent with the findings of previous studies that found that Cd stress may inhibit the growth of seedlings [37, 38]. RD did not change under Cd stress, indicating that the growth of root thickness was not affected by Cd. MRL was also less affected by Cd. In order to study the effects of Cd on rice growth, the ratios of the values under Cd stress versus the values under normal conditions (G/N) were used to measure CGRs. Considering the large genetic differences between subspecies, CGR indices among *indica*, temperate *japonica*, and tropical *japonica* rice were compared. The SW and RW indices for temperate *japonica* were higher than *indica* (*P* = 0.038 and 0.040, respectively), while the SL index for temperate and tropical *japonica* was less than *indica* (*P* = 0.0006 and 0.007, respectively). *Indica* and tropical *japonica* had similar RA (*P* = 0.59), SA (*P* = 0.66), and RV (*P* = 0.98) values, and the RV value of *indica* were lower than temperate *japonica* (*P* = 0.035). There were no differences in SRL values between subspecies (*P* > 0.05).

**Table 1:** Phenotype comparison between normal and Cd stress conditions

Traits	Full population				*Indica*			
	N	G	G/N	*P*-value	N	G	G/N	*P*-value
SRL (cm)	495.4	286.3	0.60	6.62E-42	515.9	300.6	0.60	5.22E-21
RA (cm^2^)	16.28	9.13	0.59	2.50E-41	17.17	9.51	0.57	6.75E-21
SA (cm^2^)	48.55	27.01	0.59	7.51E-41	51.24	28.07	0.57	6.34E-21
RV (cm^3^)	0.502	0.256	0.55	2.28E-36	0.531	0.259	0.52	7.14E-21
RD (mm)	0.287	0.287	1.01	8.79E-01	0.287	0.280	0.98	5.78E-02
MRL (cm)	20.80	17.76	0.86	2.30E-17	20.42	17.36	0.86	1.19E-09
SL (cm)	37.16	25.08	0.68	5.35E-50	37.08	25.76	0.70	2.05E-18
SW (g)	0.173	0.113	0.69	1.37E-21	0.186	0.119	0.66	4.16E-11
RW (g)	0.044	0.025	0.61	1.32E-29	0.047	0.063	0.57	6.73E-01
Traits	Temperate *japonica*				Tropical *japonica*			
	N	G	G/N	*P*-value	N	G	G/N	*P*-value
SRL (cm)	527.9	296.9	0.60	2.23E-15	435.0	243.5	0.58	1.31E-07
RA (cm^2^)	17.12	9.57	0.60	6.74E-15	14.53	7.90	0.56	2.35E-07
SA (cm^2^)	51.09	28.39	0.59	1.43E-14	43.29	23.40	0.56	3.07E-07
RV (cm^3^)	0.530	0.277	0.58	1.21E-11	0.463	0.229	0.52	1.58E-06
RD (mm)	0.290	0.293	1.02	6.30E-01	0.291	0.296	1.02	6.21E-01
MRL (cm)	21.19	18.29	0.87	2.24E-06	22.29	18.29	0.83	2.49E-03
SL (cm)	38.20	25.12	0.67	2.63E-20	37.22	24.44	0.66	3.48E-09
SW (g)	0.178	0.116	0.71	9.74E-09	0.150	0.098	0.71	4.16E-04
RW (g)	0.047	0.027	0.62	3.66E-10	0.038	0.023	0.65	2.50E-04

*N* normal conditions, *G* Cd stress conditions, *G/N* the ratio of the value under Cd stress conditions versus the value under normal conditions. Significant differences between normal and Cd stress conditions were analyzed by Student’s t test in R

**Fig. 1:**
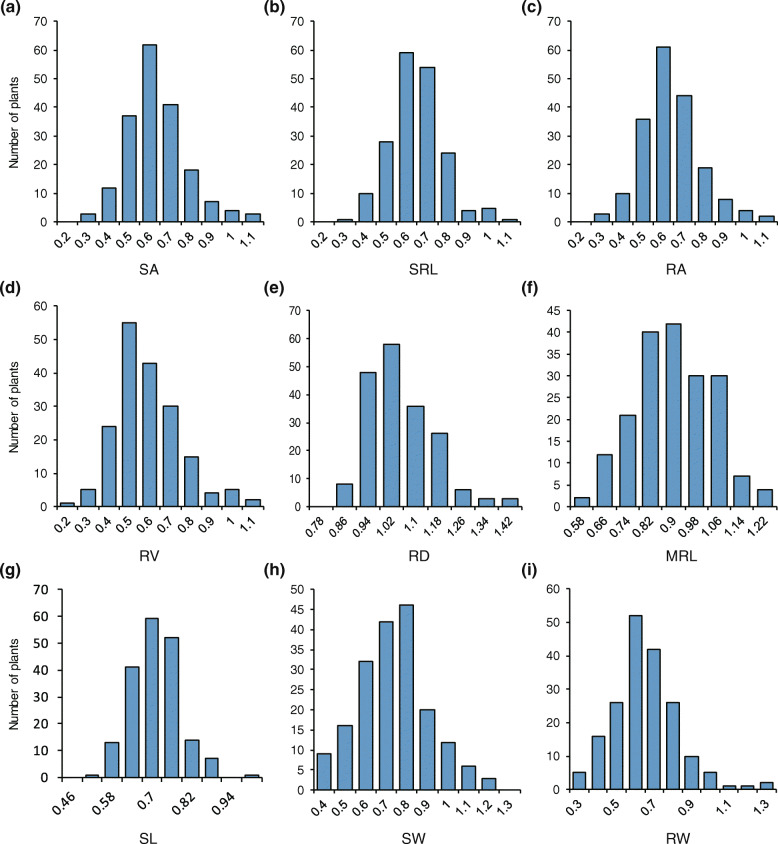
Phenotypic diversity of high Cd-mediated growth responses of different rice accessions: **a** superficial area of roots (SA), **b** sum of root length (SRL), **c** root area (RA), **d** root volume (RV), **e** root diameter (RD), **f** maximum root length (MRL), **g** shoot length (SL), **h** shoot weight (SW), and **i** root weight (RW)

The CGRs of germplasm resources exhibited a great deal of variation (Fig. 1). The variation ranges of SA, SRL, RA, RV, SW, and RW were large, while the variation ranges of RD, MRL, and SL were relatively small. High correlation coefficients were detected for SRL, RA, SA and RV indices (Fig. S1). High correlation coefficients (> 0.65) were also detected between RW and SW, RA, SA, and RV indices. Low correlation coefficients (< 0.2) were detected between RD, MRL and SL indices, suggesting that there were distinct genetic architectural differences among these indices and that a high MRL index did not indicate enhanced SL index.

### Identification of QTL for CGRs by GWAS

**Fig. 2:**
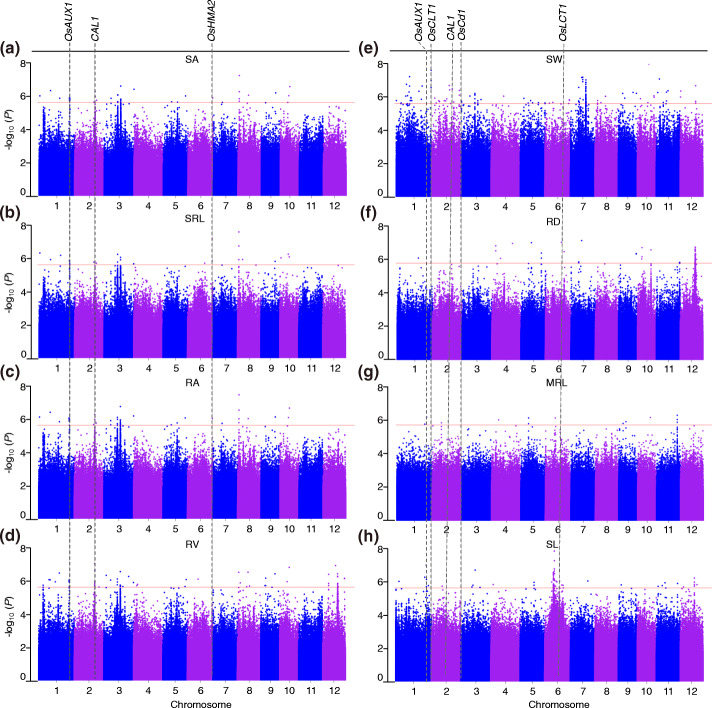
Identification of CGR QTLs by GWAS. Manhattan plots of GWAS for (**a**) SA, (**b**) SRL, (**c**) RA, (**d**) RV, (**e**) SW, (**f**) RD, (**g**) MRL, and (**h**) SL. For the Manhattan plots, -log_10_*p*-values from a genome-wide scan were plotted against the SNP positions on each of the 12 chromosomes. The horizontal light pink line indicates the suggestive threshold (*p* = 2.38×10^−6^). The grey dotted lines indicate 6 known genes that were confirmed in this study with their corresponding QTL repeatedly scanned in different traits

A GWAS was performed to identify QTL for CGR traits under an expedited mixed linear model approach (EMMAX program) based on ~3.3 million single nucleotide polymorphisms (SNPs) with missing rates of ≤ 30% and a minor allele frequency (MAF) > 0.05 covering the whole rice genome (MAF) [31, 39]. Thirteen, 9, 12, 14, 22, 8, 5, 13 and 23 CGR QTL were obtained for SA, SRL, RA, RV, SW, RD, MRL, SL and RW indices, respectively (Fig. 2, S2 and S3; Table S2). There were several common QTL intervals among the different CGR indices (Table S2). Eight common QTL were detected among SA, RA and SRL, indicating that they were pleiotropic (Table S2). There were twenty-two common QTL between two CGR indices, and 1 common QTL were detected between six CGR indices. However, there were no common QTL detected between MRL and RD indices, which was consistent with a low correlation (Fig. S1).

### Comparison of GWAS results with reported QTL/genes

**Fig. 3:**
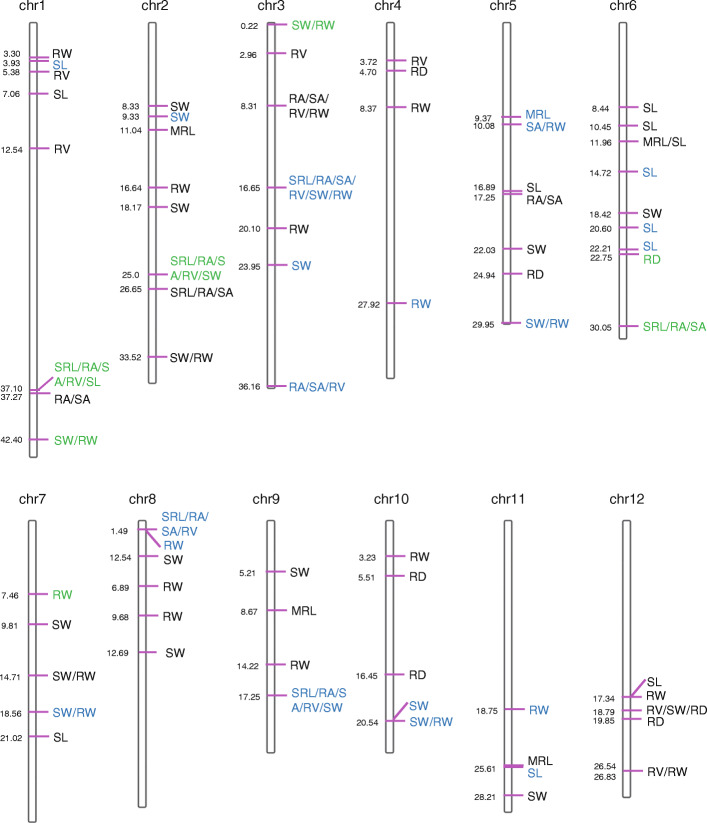
Distribution of 119 common loci on 12 chromosomes based on physical distance. Numbers on the left side of each column represent the physical location (Mb) of each lead SNP. Letters to the right of each column represent the corresponding CGR indices. QTL co-located with known QTL and genes are indicated by blue and green letters, respectively

The genomic positions of known Cd-related functional genes with the QTL intervals obtained in this study were compared. Seven genes were co-localized with the associated sites (Figs. 2 and 3; Table S2). *OsAUX1*, an auxin transporter involved in primary root and root hair elongation, and in rice Cd stress responses [24], was located in the linkage disequilibrium (LD) region where the *qSRL1.1*, *qRA1.1*, *qSA1.1*, *qRV1.3*, and *qSL1.3* loci were detected (Table S3). *CAL1* is a gene that encodes a defensin-like protein acted on by chelating Cd in the cytosol and facilitates Cd secretion to extracellular spaces. It was previously found to lower cytosolic Cd concentration, while driving long-distance Cd transport via xylem vessels [40]. In this study, *CAL1* was detected in the LD region of the *qSRL2.1*, *qRA2.1*, *qSA2.1*, *qRV2.1*, and *qSW2.4* loci. *OsHMA2*, a major transporter of Zn and Cd from roots to shoots, was located in the region of the *qSRL6.1*/ *qRA6.1*/ *qSA6.1* loci. *OsCLT1* encodes a CRT-like transporter 1 and functions as an important component of glutathione homeostasis and Cd tolerance in rice roots [20]. In this study, it was located in the region of the *qSW1.1* and *qRW1.2* loci. *OsCd1*, a major facilitator superfamily gene involved in root Cd uptake that contributes to grain accumulation in rice, was located in the region of the *qSW3.1* and *qRW3.1* loci. *OsLCT1*, a low-affinity cation transporter for phloem Cd transport in plants, was located in the region of the *qRD6.1* and *qSL6.6* loci. *OsHMA3*, a P1B-type heavy metal ATPase that transports Cd into the vacuoles for sequestration [41], was located in the region of the *qRW7.1* locus.

Moreover, the localization of associated sites detected in this study were compared with previously detected Cd-related QTL from the linkage mapping and GWAS of previous studies [14, 27, 42–46]. A total of 55 associated sites were co-localized with 28 previously reported QTL (Fig. 3; Table S2). Specifically, *qSRL3.1*, *qRA3.2*, *qSA3.2*, *qRV3.3*, and *qSW3.2* were all located in the regions of *qSRR3* (SRR, shoot/root ratio of Cd concentration) [47]; *qSRL8.1*, *qRA8.1*, *qSA8.1*, and *qRV8.1*, were located in the region of *qRDW8.2* (RDW, root dry weight); *qSRL9.1*, *qRA9.1*, and *qSA9.1*, were located in the region of *qLR-9*; *qRA3.4*, *qSA3.4*, and *qRV3.5*, were located in the region of *qCd3* (Cd, grain Cd content). These results indicate the important roles of repeatable QTL detected by linkage mapping and GWAS, and these QTL may be related to each other.

### Elite alleles in 7 cloned genes for CGR

**Fig. 4:**
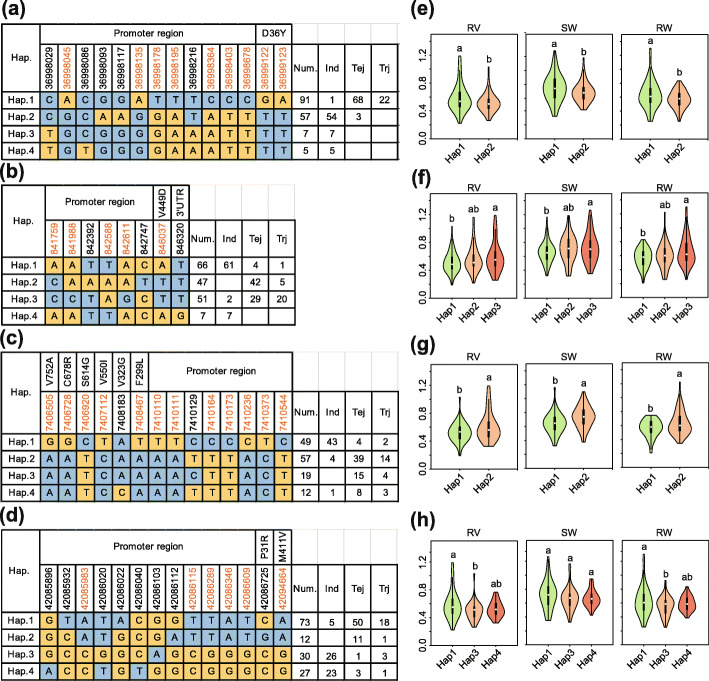
Haplotype analyses of *OsAUX1*, *OsCd1*, *OsHMA3* and *OsCLT1*. Gene structures (left) and RV, SW and RW of different haplotypes (right) of (**a**, **e**) *OsAUX1* (*LOC_Os01g63770*) for *qSRL1.1*/*qRV1.3*/*qSL1.3*, (**b**, **f**) *OsCd1* (*LOC_Os03g02380*) for *qSW3.1*/*qRW3.1*, (**c**, **g**) *OsHMA3* (*LOC_Os07g12900*) for *qRW7.1*, and (**d**, **h**) *OsCLT1* (*LOC_Os01g72570*) for *qSW1.1*/*qRW1.2*. Brown colored numbers indicate the key SNPs that occurred in major haplotypes and resulted in significant differences of CGR indices. The violin map was constructed in R. Student’s t-tests were used to determine *p-*values (*p* < 0.05). Num, number of accessions; Ind, *indica*; Tej, temperate *japonica*; trj, tropical *japonica*

To better understand the natural variation of 7 known genes, haplotype analyses were performed using all of the non-synonymous SNPs within their ORFs and promoters. Twelve SNPs in the promoter and 2 non-synonymous SNPs (i.e., Chr1_36999122 and Chr1_36999123) were detected at the *OsAUX1* locus, which play important roles in response to Cd stress and root development [24]. Four distinct haplotypes, including 2 major haplotypes, Hap.1 and Hap.2, were identified based on the 14 SNPs in cultivated rice and exhibited a large genetic difference between *indica* and *japonica* (Fig. 4a). Hap.1 was mostly present in temperate and tropical *japonica*, while Hap.2 was mostly present in *indica*. Significant differences in CGR indices (i.e., RV, SW and RW) were detected between Hap.1 and Hap.2 (Fig. 4e). Accessions with the Hap.1 genotype had higher RV, SW and RW indices than accessions of other haplotypes, indicating Cd tolerance in seedlings.

A total of 8 SNPs (MAF ≥ 0.05) in *OsCd1* identified 4 haplotypes (Fig. 4b). The vast majority of *indica* accessions were Hap.1, while *japonica* accessions were Hap.2 or Hap.3. Accessions carrying Hap.1 showed lower RV, SW and RW indices than *japonica* carrying Hap.2 or Hap.3 (Fig. 4f). Furthermore, recent studies revealed that accessions with *OsCd1*^*D449*^ have higher grain Cd concentrations compared to those with *OsCd1*^*V449*^ [14], which also exhibit a lower Cd transport ability, suggesting that the missense mutation, V449D, is responsible for the divergence of rice CGRs between *indica* and *japonica*. Interestingly, 2 *indica* accessions with higher CGR indices were Hap.3, indicating that elite *japonica* alleles had been introgressed into *indica* accessions through breeding (Fig. 4b).

Recently, Liu et al. [48] reported that variations in *OsHMA3* contributed to differential grain Cd accumulation between *indica* and *japonica*. Here, 6 non-synonymous SNPs (i.e., Chr7_7408467, Chr7_7408183, Chr7_7407112, Chr7_7406920, Chr7_7406728, and Chr7_7406505) and 8 SNPs in the promoter of *OsHMA3* revealed 2 major haplotypes, Hap.1 and Hap.2 (Fig. 4c). Hap.1 contained most *indica* rice and was associated with lower RV, SW, and RW indices. In contrast, Hap.2 contained most temperate and tropical *japonica* rice and was associated with higher RV, SW and RW indices (Fig. 4g). Similarly, 3 major haplotypes were detected in *OsCLT1* with Hap.1 was associated with higher RV, SW, and RW indices (Fig. 4d and h); its frequency was high in temperate and tropical *japonica*, but low in *indica*.

Three of 16 SNPs (i.e., Chr6_29481363, Chr6_29481366 and Chr6_29481398) in the promoter region of *OsHMA2* distinguished 2 distinct major haplotypes, Hap.1 and Hap.2 (Fig. S4a). The RV, SW and RW indices of Hap.1 were significantly higher than Hap.2 (Fig. S4d). Additionally, a premature termination codon mutation, K153* of Hap.3 was detected in *OsHMA2.* Hap.3 also had higher RV, SW and RW indices, but lower frequency.

Nine SNPs in the promoter and 1 non-synonymous SNP (i.e., Chr2_25190881) of *CAL1* revealed 3 major haplotypes (Fig. S4b). Most *japonica* carried Hap.2 and the difference in the number of *indica* rice of three major haplotypes was small. Hap.1 had higher CGR indices than Hap.3 in *indica* (Fig. S4e). However, there was no significant phenotypic difference between Hap.3 and Hap.4, indicating that the non-synonymous SNP could not be the cause of the variation affecting RV, SW and RW indices. These results suggest that the codon sequences in *CAL1* for maintaining protein function were highly conserved and that the phenotypic differences among haplotypes could be caused by differences in the expression levels of germplasm resources, which was consistent with a previous study on *CAL1* that found that it positively regulated the Cd contents in the leaves and xylem sap [13].

Only 2 haplotypes were detected by 13 non-synonymous SNPs and 10 SNPs in the promoter of *OsLCT1*. All of the *indica* accessions were belonged to Hap.2 (Fig. S4c). Both Hap.1 and Hap.2 had temperate *japonica* and tropical *japonica* rice accessions. RV and SW indices of Hap.1 were higher than Hap.2 (Fig. S4f).

### Differentially expressed rice genes in response to Cd stress from RNA-Seq data

To investigate the transcriptomic response of rice to Cd stress, an RNA-Seq analysis was performed on 3 Cd-tolerant varieties (CTVs) and 3 Cd-sensitive varieties (CSVs) under normal and Cd stress conditions (Fig. S5; Table S1). RNA-Seq data of 3 biological replicates were combined to screen the common DEGs in each variety. RNA-Seq data of 3 Cd-tolerant varieties and 3 Cd-sensitive varieties were combined to screen the common DEGs in all CTVs and CSVs, respectively. A total of 2,528 differentially expressed genes (DEGs) that respond to Cd stress were identified. More DEGs were identified in CTVs (1,068 up-regulated and 773 down-regulated genes) than CSVs (712 up-regulated and 729 down-regulated genes) under Cd stress (Fig. S5). According to the gene ontology (GO) enrichment analysis, the up-regulated DEGs in CSVs were highly enriched in oxidation reduction, zinc ion transmembrane transport, and tetrapyrrole, heme and iron ion binding, while down-regulated DEGs were enriched in oxidoreductase, carbohydrate metabolic processes, hydrolyzing O-glycosyl compounds and apoplast (Fig. S6; Table S4). Genes enriched in the GO terms oxidation reduction, aminoglycan catabolic process, heme binding and iron ion binding were up-regulated, while genes enriched in the GO terms photosynthesis, oxidoreductase activity, thylakoid and photosynthetic membrane were down-regulated in CTVs (Fig. S7; Table S5). Interestingly, the down-regulated genes in CSVs enriched in hydrolase activity, hydrolyzing O-glycosyl compounds, endopeptidase inhibitor activity, and peptidase inhibitor activity were up-regulated in CTVs (Figs. S6 and S7).

**Fig. 5:**
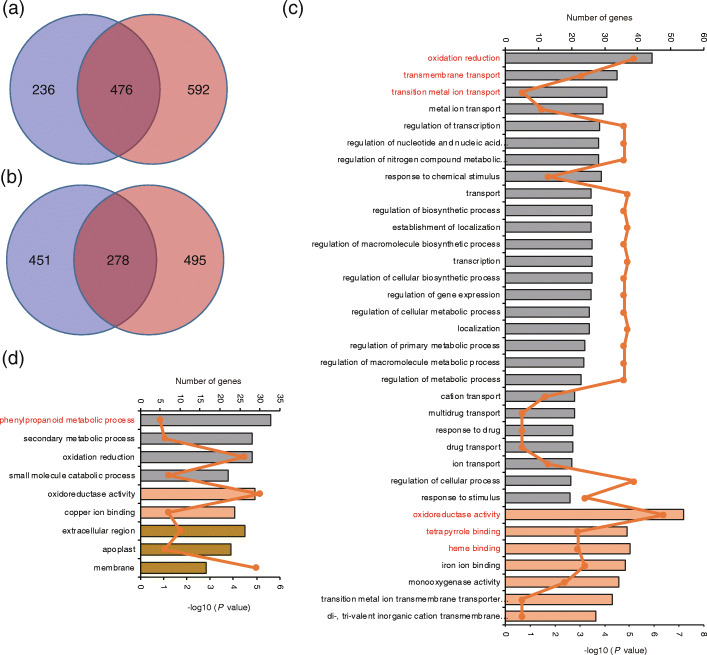
Genome-wide transcriptome analysis of Cd stress between Cd-tolerant and Cd-sensitive rice varieties: modified Venn diagrams showing the common and specific (**a**) up-regulated and (**b**) down-regulated genes in response to Cd stress; GO enrichment analysis of genes that were (**c**) co-up-regulated and (**d**) co-down-regulated in response to Cd stress among CSVs and CTVs. GO terms mentioned in the text are highlighted in red. Histograms indicate the -log_10_*p*-value. Broken lines indicate the number of genes

There were 476 co-up-regulated and 278 co-down-regulated genes among CTVs and CSVs (Fig. 5a and b; Table S6). The GO analysis revealed that these common genes were enriched in oxidation reduction, tetrapyrrole binding, heme binding, metal ion transmembrane transport, and phenylpropanoid metabolic processes (Fig. 5c and d). For CTV-specific DEGs, up-regulated genes were enriched in oxidation reduction, iron ion binding, and heme binding, while down-regulated genes were enriched in photosynthesis, thylakoid, and photosynthetic membrane (Fig. S8; Table S7). Chitin has selective permeability in material transport and plays a role in attracting cations [49–52]. Interestingly, genes enriched in the GO term, chitin metabolic process, were up-regulated in CTVs (Fig. S8), suggesting that chitin may be associated with CGRs in CTVs. The term oxidation reduction in biological process and the term oxidoreductase activity in molecular function were the most significant GO terms in CTV-specific up-regulated terms and they contained 29 cytochrome P450, 8 peroxidase precursor, 7 dehydrogenase, 4 flavin-containing monooxygenase family protein, and 3 NADP-dependent oxidoreductase genes, which could help to reduce reactive oxygen species (ROS)-relevant oxidative damage (Fig. S8; Table S7). The terms photosynthesis in biological process and the term membrane in cellular component in CTV-specific down-regulated terms contained 25 genes related to photosynthesis and 13 transporters genes, which may function in plant growth and Cd transport.

### Determination of candidate genes within CGR QTL by integrated genomic and transcriptomic analyses

The CGR loci identified by GWAS provided important clues for understanding the genetic architecture of the observed variations in rice growth under Cd stress. To identify the candidate genes responsible for each CGR locus, based on the LD decay values in *indica* and *japonica* (123 kb and 167 kb, respectively), all of the genes within 200 kb of the most significant SNPs were extracted and the data derived from the RNA-Seq analysis, the gene ontology (GO) Slim analysis [14], and their annotations and functions of homologous genes were considered. Firstly, there were 1,594 genes in intervals of 119 CGR QTL, including 921 clearly annotated genes (Table S8). Eighty-eight candidate genes from 74 CGR QTL were obtained by the integrated genomic and transcriptomic analyses (Table S9). Enriched GO terms of common genes between the GWAS and RNA-Seq analysis included response to stimulus (GO:0050896), transporter activity (GO:0005215), electron carrier activity (GO:0009055), and iron ion binding (GO:0005506) (Fig. S9). Secondly, in order to identify Cd-related membrane transporters in rice, a GO Slim analysis was conducted and the genes related to membrane and transport were selected as important candidate genes. Altogether, 53 candidate genes from 48 CGR QTL located on 12 chromosomes were found to be associated transport and membrane (Fig. S10; Table S10). Thirdly, according to the function of gene annotations and homologous genes, 12 candidate genes in 13 QTL intervals were identified (Table S11). By applying these approaches, a total of 148 genes from 85 CGR QTL were identified, being selected as potential candidate genes for each of the loci controlling CGR traits in rice (Table S11). Among them, 7 cloned Cd-related genes (i.e., *OsCd1*, *OsAUX1*, *OsHMA2*, *OsHMA3*, *OsCLT1*, *CAL1* and *OsLCT1*) were identified for the CGR genes. More importantly, some reported genes also identified in this study (i.e., *OsATX1*, *OsBOR3*, *OsTIP2*, *OsZIP8*, *OsVAMP714*, *OsHMA5*, *OsCAX1b*, *QHB*, *OsABI5*, *UbL402*, *OsGA20ox1/qEPD2/GNP1* and *OsUGE1*) were found to play certain roles in CGRs.

### Haplotype analyses of 6 QTL genes for CGRs

Among the closely associated candidate genes, several reported genes had no evidence regarding their functions in the control of CGR traits. Six of these genes (i.e., *OsGSTU31* (*LOC_Os10g38189*), *OsGSTU6.1* (*LOC_Os10g38340*), *OsGSTU6.2* (*LOC_Os10g38360*), *OsGSTU21* (*LOC_Os10g38150*), *OsGSTU32* (*LOC_Os10g38314*), and *OsATX1*) were selected for functional analyses as case studies.

**Fig. 6:**
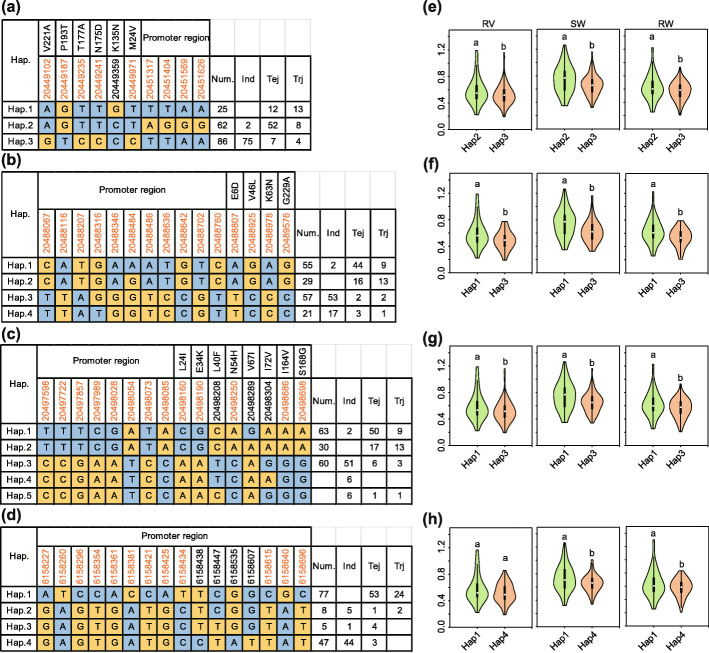
Haplotype analyses of 4 novel QTL genes for CGRs. Gene structures (left) and RV, SW, and RW of different haplotypes (right) of (**a**, **e**) *OsGSTU31* (*LOC_Os10g38189*) for *qSW10.2*/*qRW10.2*, (b, f) *OsGSTU6.1* (*LOC_Os10g38340*) for *qSW10.2*/*qRW10.2*, (**c**, **g**) *OsGSTU6.2* (*LOC_Os10g38360*) for *qSW10.2*/*qRW10.2*, and (**d**, **h**) *OsATX1* for *qSRL8.2*. Brown colored numbers indicate the key SNPs that occurred in major haplotypes and resulted in significant differences of CGR indices. Student’s t-tests were used to determine *p-*values (*p* < 0.05)

One QTL (i.e., *qSW10.2/qRW10.2*) controlling both SW and RW on chromosome 10 was identified by GWAS and 38 genes were in the interval. Among them, 5 candidate genes, all encoding glutathione S-transferase (GST), were up-regulated under Cd stress according to the transcriptomic data (Table S11). Using the MSU Rice Genome Annotation (Osa1) Release 7 for annotated genes, 20 GST genes in or around this QTL interval were identified, 12 of which were co-up-regulated under Cd stress (Tables S4 and S5). The tolerance of plant cells to toxic elements is highly dependent on glutathione metabolism. First, GST proteins indirectly act on Cd accumulated reactive oxygen species (ROS) by maintaining the antioxidant flavonoid pool [53]. GSTs detoxify cytotoxic substrates and ameliorate their toxicity by catalyzing the conjugation of glutathione to substrates [54]. These findings suggest that this gene cluster could play an important role in the CGRs of rice. Further investigations were conducted to analyze the haplotypes of 5 GST genes within this QTL. Four SNPs in the promoter and 6 non-synonymous SNPs (i.e., Chr10_20449102, Chr10_20449187, Chr10_20449235, Chr10_20449241, Chr10_20449359, and Chr10_36999971) were detected in *OsGSTU31*. Three distinct haplotypes, including 2 major haplotypes, Hap.2 and Hap.3, were identified based on the 10 aforementioned SNPs and exhibited large genetic differences between *indica* and *japonica* (Fig. 6a). Hap.2 contained most temperate and tropical *japonica*, while Hap.3 contained the most *indica*. Significant differences in CGR indices were detected between Hap.2 and Hap.3 (Fig. 6e). Accessions with the Hap.2 genotype had higher RV, SW, and RW indices than accessions of other haplotypes and exhibited Cd tolerance in seedlings. Four non-synonymous SNPs and 11 SNPs in the promoter of *OsGSTU6.1* revealed 2 major haplotypes, Hap.1 and Hap.3 (Fig. 6b). Hap.1 contained most *japonica* and was associated with higher RV, SW, and RW indices. In contrast, Hap.3 contained most *Indica* and was associated with lower RV, SW, and RW indices (Fig. 6f). Similarly, 2 major haplotypes were detected in *OsGSTU6.2*, *OsGSTU21*, and *OsGSTU32* with Hap.1, Hap.3, and Hap.2 associated with higher RV, SW, and RW values, respectively (Fig. 6c, g and S11). Their frequency was high in temperate and tropical *japonica*, but low in *indica*.

There were 14 annotated genes in the *qSRL8.2* QTL interval (Table S5). A heavy metal-associated domain containing protein, *OsATX1* (*LOC_Os08g10480*), was detected, which was reported to exhibit the heterologous expression of *OsATX1* in a Cd-sensitive mutant of yeast (*Saccharomyces cerevisiae*), Δycf1, which increased the tolerance to Cd by decreasing their respective concentrations in transformed yeast cells [55]. Interestingly, no polymorphism was detected in the code region of *OsATX1* among rice accessions, suggesting that the sequences in *OsATX1* for maintaining protein function were highly conserved. Twelve SNPs in the promoter region of *OsATX1* revealed 2 major haplotypes and Hap.1 was predominant and associated with higher RV, SW, and RW indices than Hap.4 (Fig. 6d, h). These results suggest that phenotypic differences among different haplotypes could be caused by differences in the expression levels of *OsATX1* among rice accessions.

## Discussion

The growth and development of rice are inhibited under Cd stress. Whether low grain Cd rice or high shoot Cd rice varieties for phytoremediation, both of these varieties were required to grow well under Cd stress. Using rice mini-core germplasms to systematically study the differences in rice growth responses to Cd, Cd-tolerant varieties and Cd-sensitive varieties were screened in this study in order to identify Cd-tolerant genes. To the best of our knowledge, this was the first attempt to conduct a GWAS for CGR traits. In total, 119 QTL for CGRs were identified, 55 of which overlapped with previously reported Cd-related QTL. Based on an integrated analysis strategy, a total of 148 candidate genes for CGRs, including 7 cloned Cd-related genes, were identified. Elite alleles of 13 genes were investigated and will serve as potential candidates for the genetic improvement of Cd-tolerant rice.

### The complexity of genetic control of CGR traits and exploration of candidate genes

Cd is a toxic heavy metal that inhibits the growth of roots and shoots, reduces leaf and tiller number, physiologically impairs photosynthesis and mitochondrial respiration, and results in DNA degradation and cell death [44, 56]. Various traits exhibited different responses to Cd. The SRL, RA, SA, RV, and RW were considerably different between normal and Cd stress conditions, while RD and MRL exhibited minor change (Table 1). Different responses to Cd were detected among accessions from different subgroups. Both temperate and tropical *japonica* had higher RW and SW indices than *indica*, while RV index of *indica* and tropical *japonica* were lower than temperate *japonica* (Table 1). In this study, > 100 QTL were identified for CGR traits (Table S2), suggesting that the genetic regulation of CGRs is very complex and many genes play an important role in the growth of rice under Cd stress. However, only 3 Cd-related QTL (i.e., *OsHMA3*, *CAL1*, and *OsCd1*) were cloned. The findings of this study provide important information for cloning novel CGR genes in future studies.

Considering the relatively low LD decay of rice, 1 associated locus in this study was defined as a 200 kb region containing > 10 genes [57]; therefore, it was rather difficult to pinpoint the causal genes for these loci. However, the combined use of QTL information, expression profiles, GO Slim analysis, and prediction of gene functions could help uncover candidate genes, just as candidate genes were uncovered in this study. Based on the RNA-Seq data, GO Slim analysis, and gene annotations, 88 candidate genes for 74 CGR QTL, 53 for 48 CGR QTL, and 12 for 13 CGR QTL were identified (Tables S5, S6, and S7). Furthermore, many known genes were located in these CGR QTL. Collectively, this study provided a relatively comprehensive analysis of the genetic architecture of CGR in rice.

### Favorable natural haplotypes/alleles for the improvement of Cd tolerance in rice

The mining of more favorable alleles of CGR genes is required in order to achieve ideal Cd tolerance in rice. At the single-gene level, favorable haplotypes for 13 CGR genes were identified (Figs. 4 and 6, S4, and S11), including 7 known Cd-related genes and 6 novel genes. Most *japonica* accessions carried superior *OsHMA3*^*Hap2*^, *OsHMA2*^*Hap1*^, *OsCLT1*^*Hap1*^, *OsCd1*^*Hap3*^, *OsAUX1*^*Hap1*^*, OsATX1*^*Hap1*^, *LOC_Os10g38150*^*Hap1*^*, LOC_Os10g38189*^*Hap2*^, *LOC_Os10g38314*^*Hap2*^*, LOC_Os10g38340*^*Hap1*^, and *LOC_Os10g38360*^*Hap1*^ haplotypes. Additionally, elite *japonica* alleles of these genes can be considered as primary alternatives for improving Cd-tolerance in *indica*. Because the expression of *OsHMA3*, *CAL1*, and *OsAUX1* were induced by Cd [13, 24, 41, 48], natural variations in the promoter region of rice accessions were likely important functional SNPs associated with CGR traits. The results of the haplotype analysis indicated that major haplotypes, which consist of non-synonymous SNPs in the coding sequence (CDS) and/or promoter regions within single loci, represent important allelic diversity of QTL underlying the variation of CGRs in rice populations.

OsHMA3 is a cadmium transporter located in the vacuolar membrane of rice roots, belonging to the heavy metal ATPase (HMA) family. OsHMA3 could transport Cd^2+^ into vacuoles to isolate it and reduce the transport of Cd^2+^ to the aboveground, thus reducing cadmium toxicity [41, 58]. Four haplotypes were identified in rice diversity populations. There were five non synonymous SNPs and 8 SNPs on the promoter between haplotype 1 and 2 (Fig S4). Among them, F229L, V323G were located in A-Domain, V550I, S614G, C678R in ATP- binding domain, and V752A in Metal- binding domain of OsHMA3 (Yan et al. 2016). The SNPs in the promoter had been proved to affect the transcriptional activity [48]. Therefore, the differential functions of *OsHMA3* between Hap.1 and Hap.2 could be attributed to the eight nucleotide changes occurring in the promoter region. OsHMA2 is a major Zn and Cd transporter in rice roots and shoots. It is homologous with OsHMA3, a heavy metal ATPase. Its C-terminal region is essential for Cd transport in shoot [11]. In rice accessions, Hap.1 had significantly higher RV, SW and RW indices than Hap.2, indicating that the three SNPs (i.e., Chr6_29481363, Chr6_29481366 and Chr6_29481398) could play important role in response to Cd for rice. Hap.3 of *OsHMA2* had a premature termination codon mutation and resulted in a truncated protein product, which could affect its cadmium transport function. Ten *indica* rice and 4 temperate *japonica* rice in Hap.3 were primary improved varieties, only one was recently improved varieties (Fig S4g and Table S12). Compared with Hap.1 and Hap.2, Hap.3 had relatively fewer varieties. These results suggested that Hap.3 was just found recently and had already been used in recent rice breeding, but it was rarely used at present. Future breeding with Hap. 3 is a potential alternative for improving Cd tolerance in current elite varieties.

### Natural variations in 6 QTL genes served important roles in CGR

*ATX1* of Saccharomyces cerevisiae encodes an 8.2-kDa peptide, which has significant similarity with many bacterial metal transporters. ATX1 is involved in the transport and distribution of copper and protects cells from the toxicity of superoxide anion and hydrogen peroxide [59]. The resistance of Saccharomyces cerevisiae atx1 deletion strain to Cd^2+^ was higher than that of wild type and ATX1 can specifically bind Cd^2+^ [60]. The two copper chaperones of *Arabidopsis thaliana*, namely Antioxidant Protein1 (ATX1) and ATX1-Like Copper Chaperone (CCH) (CCH), share high sequence homology [61]. Arabidopsis *Antioxidant Protein1* (*ATX1*) plays an essential role in copper (Cu) homeostasis, conferring tolerance to both excess and subclinically deficient Cu [62]. The high affinity of Cd for thiols might be the reason that Cd^2+^ also can bind the Cu-binding motif MXCXXC, which was required for the physiological function of ATX1 [61]. Knockout of *OsATX1* resulted in an increase of Cu concentration in roots, while overexpression of *OsATX1* decreased root Cu concentration but increased shoot Cu accumulation. The concentrations of Cu in developing tissues, including upper nodes and internodes, younger leaf blades, and leaf sheaths, were increased significantly in *OsATX1*-overexpressing plants and decreased in *osatx1* mutants compared with the wild type [61], indicating that rice varieties with high *OsATX1* expression might show more sensitive to Cd. In fact, overexpression of *OsATX1* increased Cd accumulation in the shoots [55]. Haplotype analysis showed that *OsATX1* showed significant *indica-japonica* differentiation. The high RV, SW, and RW indices of *japonica* rice might be resulted from their low *OsATX1* expression (Fig S12). Thus, gene editing of *OsATX1* promoter could improve cadmium tolerance of cultivated rice.

The induction of oxidative stress by Cd was one of the major alterations in plant cells [56]. When redox imbalance occurs, membrane lipids, proteins, and nucleic acids were oxidized, which in turn affects plant metabolism. Glutathione functioned as an antioxidant and moderated the redox imbalance induced by toxic metal accumulation in *Arabidopsis* [63]. Moreover, *OsGST4* played an important role during oxidative stress by ROS-scavenging in rice [64]. In this study, the RNA-Seq data revealed that many genes enriched in the GO term, oxidation reduction, responded to Cd stress (Fig. 4c and d). Cd may be formed as a complex with phytochelatins or glutathione, and is subsequently transported to the vacuoles through an unidentified ABC transporter. Glutathione is used to detoxify xenobiotics through GSTs. GST family genes were previously found to play roles in Cd resistance and accumulation of pak choi [65]. In this study, a GST gene cluster on chromosome 10 was identified by the integrated genomic and transcriptomic analyses (Table S11). Twelve of these genes were up-regulated under Cd stress. The haplotype analysis revealed that all 5 *OsGSTUs* in the QTL interval showed *indica*-*japonica* differentiation and their *japonica* haplotypes had higher CGR indices (Figs. 6a–c and S11). These results suggest that the GST gene cluster played an active role in detoxification and the *japonica* alleles of the 5 GSTs enabled rice to grow better under Cd stress.

## Conclusions

The CGRs of germplasm resources exhibited a great deal of variation and the influence of Cd on the growth of *indica* rice was greater than that of *japonica* rice. A total of 148 genes from 85 CGR QTL were obtained by comprehensive analyses. Natural elite haplotypes/alleles of 13 genes, including 7 cloned Cd-related genes and 6 novel genes, are identified and will serve as potential candidates for the genetic improvement of Cd-tolerant rice. The cultivation of novel Cd-tolerant varieties also helps to ensure a stable rice yield.

## Methods

### Plant materials and phenotyping

A total of 188 rice accessions from around the world were used for evaluating CGRs in this study. Hydroponic experiments were performed at the greenhouse of Agricultural Genomics Institute in Shenzhen, China during the summer of 2018. All of the seeds were soaked in deionized water at 37°C in the dark for 2 d, then transferred to a net floating on deionized water for 5 d. Seedlings were cultured in a half-strength Kimura B nutrient solution (pH, 5.4) with the following composition (μM): 90 KH_2_PO_4_, 270 MgSO_4_, 180 (NH_4_)_2_SO_4_, 90 KNO_3_, 180 Ca(NO_3_)_2_, 3 H_3_BO_3_, 0.5 MnCl_2_, 1 (NH_4_)_6_Mo_7_O_24_, 0.4 ZnSO_4_, and 20 Fe(III)-EDTA. Solutions were changed 3 times per week and the pH was adjusted to 5.4 every day. Plants were grown in a greenhouse with natural sunlight at 30°C during the day and 25°C at night [48]. In order to compare the growth of normal and Cd-treated seedlings, 15-d-old plants were exposed to Cd stress in a 1/2 Kimura B nutrient solution containing 50 μM CdCl_2_ for 7 d, solutions were renewed every 2 d. The experiment was conducted three times. Five plants per variety were sampled and the sum of root length (SRL), root area (RA), superficial area of root (SA), root volume (RV), root diameter (RD), maximum root length (MRL), shoot length (SL), shoot weight (SW), and root weight (RW) were measured.

### GWAS

The sequence data of all of the rice accessions for GWAS were obtained from the 3,000 Rice Genomes Project [66]. The SNP data were filtered out with minor allele frequencies (MAF) < 0.05 and missing rates > 30%. The efficient mixed model analysis (EMMA) feature of the EMMA eXpedited (EMMAX) software was employed for GWAS [39]. The significance threshold was calculated using the formula “-log10(1/the effective number of independent SNPs)” as described previously [67], and effective numbers of independent SNPs were determined by PLINK to be 398107 in this population [68]. The suggestive *P* values was 2.5 × 10^−6^. Finally, the threshold was set at −log(*P*) = 5.6 to identify significant association signals. Based on the LD decay values in *indica* and *japonica* rice (123 kb and 167 kb, respectively), several SNPs passing the threshold on the same chromosome were clustered as one associated locus with a region of < 200 kb. All genes located within the candidate region were extracted [63].

### RNA-Seq and GO enrichment analyses

Three Cd-tolerance cultivars (CTVs) (CX47, Yungeng 23 and IRIS_313_9050) and 3 Cd-sensitive cultivars (CSVs) (GUI630, ALBANIA_SPECIES and BAXIANG) based on phenotyping results were selected for the RNA-Seq analysis (Fig. S5; Table S1). 15-d-old plants of the 6 varieties were planted under normal and Cd stress conditions for 12 h, and then the roots were sampled for RNA extraction. RNA was extracted by preparing samples using a Micro RNA Extraction kit (Axygen, NY, USA) and reverse transcribed into cDNA using a ReverTra® Ace qPCR-RT kit (TOYOBA, Osaka, Japan). RNA-Seq libraries were prepared using 3 biological replicates for each variety and sequenced separately using a Hiseq Xten sequencer. TOPhat2 software [69] was used to align the cleanup data to the reference genome MSU V7.0 [70] and gene expression was quantified by fragment per kilobase million (FPKM) using the Cufflinks [69] default parameters. A false discovery rate (FDR) < 0.05 and absolute value of log_2_ ratio ≥ 1 were used to identify differentially expressed genes (DEGs) as previously described [71]. GO enrichment analyses were conducted using agriGO v2, an online GO analysis toolkit and database for agricultural communities [72].

### Statistical analysis

Correlation coefficients between the measured traits were calculated using the R package PerformanceAnalytics [73] as described in Note S1. The violin map for haplotype analysis was also constructed in R. Data were statistically analyzed and multiple comparisons were made using Duncan’s multiple range test as described [74]. *P* values of less than 0.05 were considered to indicate statistical significance. Different letters denote significant differences. Statistical calculations were performed using Microsoft Excel 2010.

## Supplementary Information

**Additional file 1: Fig. S1.** Correlation coefficient among 9 traits related to high cadmium-mediated growth responses.

**Additional file 2: Fig S2.** Quantile-quantile plot for SA (a), SRL (b), RA (c), RV (d), SW (e), RD (f), MRL (g), and SL (h).

**Additional file 3: Fig. S3.** Identification of RW QTLs for cadmium-mediated growth responses by GWAS.

**Additional file 4: Fig. S4.** Haplotype analysis of *OsHMA2*, *CAL1* and *OsLCT1*.

**Additional file 5: Fig. S5.** Modified Venn diagrams showing the common and specific Cd-responsive genes across different Cd-tolerant and Cd-sensitive rice varieties.

**Additional file 6: Fig. S6.** GO enrichment analysis of DEGs in response to Cd stress in CSVs.

**Additional file 7: Fig. S7.** GO enrichment analysis of DEGs in response to Cd stress in CTVs.

**Additional file 8: Fig. S8.** GO enrichment analysis of specific Cd-responsive genes in CTVs.

**Additional file 9: Fig. S9.** GO enrichment analysis of common genes between GWAS and RNA-seq.

**Additional file 10: Fig. S10.** The numbers of genes annotated with transport and membrane from the Go Slim assignments for annotated genes.

**Additional file 11: Fig. S11.** Haplotype analyses of two QTL genes for cadmium-mediated growth responses.

**Additional file 12: Fig. S12.** Expression analyses of *OsATX1* in natural rice varieties.

**Additional file 13: Table S1.** Information about 188 accessions utilized in our study.

**Additional file 14: Table S2.** Summary of QTLs for cadmium-mediated growth responses by GWAS.

**Additional file 15: Table S3.** List of 7 reported cadmium related genes in regions of association loci.

**Additional file 16: Table S4.** DEGs in response to Cd stress in CSVs.

**Additional file 17: Table S5.** DEGs in response to Cd stress in CTVs.

**Additional file 18: Table S6.** The common Cd-responsive genes across different Cd-tolerant and Cd-sensitive rice varieties.

**Additional file 19: Table S7.** The specific Cd-responsive genes in CTVs.

**Additional file 20: Table S8.** The list of 921 well-annotated genes in CGR QTL intervals.

**Additional file 21: Table S9.** Determination of 88 candidate genes of CGR QTLs by integrated GWAS and transcriptomic analyses.

**Additional file 22: Table S10.** The 53 candidate genes and the Go Slim assignments for annotated genes in QTLs

**Additional file 23: Table S11.** Determination of candidate genes for CGR QTLs by integrated genomic, transcriptomic analyses, GO Slim analysis and gene annotations.

**Additional file 24: Table S12.** Documented variety type information of 16 varieties in Hap.3 of OsHMA2.

**Additional file 25.**

## Data Availability

Gene annotation referred to RGAP (http://rice.plantbiology.msu.edu/). All of the SNP data were obtained from the Rice Functional Genomics and Breeding (RFGB) database version 2.0 (http://www.rmbreeding.cn/Index/). SNP and Genotype data for GWAS can be downloaded from 3K Rice Genomes Project database (https://snpseek.irri.org/_download.zul). Information about 188 accessions utilized in our study can be found in Table S1. We extracted 188 accessions SNP genotype from 3K Rice SNP database. Reference genome MSU V7.0 was used (http://rice.plantbiology.msu.edu/pub/data/Eukaryotic_Projects/o_sativa/annotation_dbs/pseudomolecules/). Transcriptome information from this research can be downloaded in the NCBI Sequence Read Archive (http://www.ncbi.nlm.nih.gov/sra) through accession number PRJNA745371. Other datasets supporting the conclusions of this article are included within the article and its additional files.

## Abbreviations

Cd: Cadmium
SNPs: Single nucleotide polymorphisms
CGR: Cd-mediated growth response
QTL: Quantitative trait loci
GWAS: Genome-wide association study
GO: Gene ontology
EMMA: Efficient mixed model analysis
SRL: Root length
RA: Root area
SA: Superficial area of root
RV: Root volume
RD: Root diameter
MRL: Maximum root length
SL: Shoot length
SW: Shoot weight
RW: Root weight
CTVs: Cd-tolerance cultivars
CSVs: Cd-sensitive cultivars
FDR: False discovery rate
DEGs: Differentially expressed genes
MAF: Minor allele frequency
GST: Glutathione S-transferase
ROS: Reactive oxygen species

## References

[1] Moreno-Caselles M, Perez-Espinosa, Perez-Murcia MD. Cadmium accumulation and distribution in cucumber plant. J Plant Nutr. 2000;23(2):243-50.

[2] Moriarty F. Ecotoxicology: The study of pollutants in ecosystems. J Wildlife Manage. 1999;48(4):1465.

[3] Uraguchi S, Fujiwara T (2012). Cadmium transport and tolerance in rice: perspectives for reducing grain cadmium accumulation. Rice.

[4] Seregin IV, Ivanov VB (2001). Physiological aspects of cadmium and lead toxic effects on higher plants. Russ J Plant Physiol..

[5] VAN Assche F, Clijsters H (1990). Effects of metals on enzyme activity in plants. Plant Cell Environ..

[6] Clemens S (2006). Toxic metal accumulation, responses to exposure and mechanisms of tolerance in plants. Biochimie.

[7] Nakanishi H, Ogawa I, Ishimaru Y, Mori S, Nishizawa NK (2006). Iron deficiency enhances cadmium uptake and translocation mediated by the Fe^2+^ transporters OsIRT1 and OsIRT2 in rice. Soil Sci Plant Nutr..

[8] Takahashi R, Ishimaru Y, Nakanishi H, Nishizawa NK (2011). Role of the iron transporter OsNRAMP1 in cadmium uptake and accumulation in rice. Plant Signal Behav..

[9] Sasaki A, Yamaji N, Yokosho K, Ma JF (2012). *Nramp5* is a major transporter responsible for manganese and cadmium uptake in rice. Plant Cell.

[10] Ueno D, Yamaji N, Kono I, Huang CF, Ando T, Yano M, Ma JF (2010). Gene limiting cadmium accumulation in rice. Proc Natl Acad of Sci U S A..

[11] Satoh-Nagasawa N, Mori M, Nakazawa N, Kawamoto T, Nagato Y, Sakurai K, Takahashi H, Watanabe A, Akagi H (2012). Mutations in rice (Oryza *sativa*) *heavy metal ATPase 2* (*OsHMA2*) restrict the translocation of zinc and cadmium. Plant Cell Physiol..

[12] Uraguchi S, Kamiya T, Sakamoto T, Kasai K, Sato Y, Nagamura Y, Yoshida A, Kyozuka J, Ishikawa S, Fujiwara T (2011). Low-affinity cation transporter (OsLCT1) regulates cadmium transport into rice grains. Proc Natl Acad of Sci U S A..

[13] Luo JS, Huang J, Zeng DL, Peng JS, Zhang GB, Ma HL, Guan Y, Yi HY, Fu YL, Han B (2018). A defensin-like protein drives cadmium efflux and allocation in rice. Nat Commun..

[14] Yan H, Xu W, Xie J, Gao Y, Wu L, Sun L, Feng L, Chen X, Zhang T, Dai C (2019). Variation of a major facilitator superfamily gene contributes to differential cadmium accumulation between rice subspecies. Nat Commun..

[15] Liu XS, Feng SJ, Zhang BQ, Wang MQ, Cao HW, Rono JK, Chen X, Yang ZM (2019). OsZIP1 functions as a metal efflux transporter limiting excess zinc, copper and cadmium accumulation in rice. BMC Plant Biol..

[16] Shimo H, Ishimaru Y, An G, Yamakawa T, Nakanishi H, Nishizawa NK (2011). *Low cadmium* (*LCD*), a novel gene related to cadmium tolerance and accumulation in rice. J Exp Bot..

[17] Yadav AK, Shankar A, Jha SK, Kanwar P, Pandey A, Pandey GK (2015). A rice tonoplastic calcium exchanger, OsCCX2 mediates Ca^2+^/cation transport in yeast. Sci Rep..

[18] Das N, Bhattacharya S, Bhattacharyya S, Maiti MK (2017). Identification of alternatively spliced transcripts of rice phytochelatin synthase 2 gene *OsPCS2* involved in mitigation of cadmium and arsenic stresses. Plant Mol Biol..

[19] Li JC, Guo JB, Xu WZ, Ma M (2010). RNA interference-mediated silencing of phytochelatin synthase gene reduce cadmium accumulation in rice seeds. J Integr Plant Biol..

[20] Yang J, Gao MX, Hu H, Ding XM, Lin HW, Wang L, Xu JM, Mao CZ, Zhao FJ, Wu ZC (2016). OsCLT1, a CRT-like transporter 1, is required for glutathione homeostasis and arsenic tolerance in rice. New Phytol..

[21] Shimpei H. Masato, Kuramata, Tadashi, Abe, Hiroki, Takagi, Kenjirou, Ozawa: Phytochelatin synthase OsPCS1 plays a crucial role in reducing arsenic levels in rice grains. Plant J. 2017;91(5):840-8. 10.1111/tpj.13612 28621830

[22] Shimpei U, Nobuhiro T, Christian H, Kaho A, Naoko O-O. Phytochelatin Synthase has Contrasting Effects on Cadmium and Arsenic Accumulation in Rice Grains. Plant Cell Physiol. 2017;58(10):1730-42. 10.1093/pcp/pcx114 PMC5914395 29016913

[23] Shinichi Y, Yosuke U, Aya M, Kumiko O, Toru M. Rice phytochelatin synthases OsPCS1 and OsPCS2 make different contributions to cadmium and arsenic tolerance. Plant Direct. 2018;2(1):e00034. 10.1002/pld3.34 PMC6508543 31245682

[24] Yu C, Sun C, Shen C, Wang S, Liu F, Liu Y, Chen Y, Li C, Qian Q, Aryal B (2015). The auxin transporter, OsAUX1, is involved in primary root and root hair elongation and in Cd stress responses in rice (Oryza sativa L.). Plant J..

[25] Li X, Zhang X, Li B, Wu Y, Sun H, Yang Y (2017). Cadmium phytoremediation potential of turnip compared with three common high Cd-accumulating plants. Environ Sci Pollut R..

[26] Santos MS, Pedro CA, Goncalves SC, Ferreira SM (2015). Phytoremediation of cadmium by the facultative halophyte plant Bolboschoenus maritimus (L.) Palla, at different salinities. Environ Sci Pollut R..

[27] Xiuyan L, Sunlu C, Mingxue G, Zheng Y, Peng X. Association Study Reveals Genetic Loci Responsible for Arsenic, Cadmium and Lead Accumulation in Rice Grain in Contaminated Farmlands. Front Plant Sci. 2019;10:61. 10.3389/fpls.2019.00061 PMC6370710 30804959

[28] Wang Q, Zeng X, Song Q, Sun Y, Lai Y. Identification of key genes and modules in response to Cadmium stress in different rice varieties and stem nodes by weighted gene co-expression network analysis. Sci Rep. 2020;10(1):9525. 10.1038/s41598-020-66132-4 PMC7293223 32533096

[29] Huang X, Wei X, Sang T, Zhao Q, Feng Q, Zhao Y, Li C, Zhu C, Lu T, Zhang Z (2010). Genome-wide association studies of 14 agronomic traits in rice landraces. Nat Genet..

[30] Yano K, Yamamoto E, Aya K, Takeuchi H, Lo PC, Hu L, et al. Genome-wide association study using whole-genome sequencing rapidly identifies new genes influencing agronomic traits in rice. Nat Genet. 2016;48(8):927-34. 10.1038/ng.3596 27322545

[31] Yu J, Xiong H, Zhu X, Zhang H, Li H, Miao J, Wang W, Tang Z, Zhang Z, Yao G (2017). OsLG3 contributing to rice grain length and yield was mined by Ho-LAMap. BMC Biol..

[32] Zhao K, Tung C-W, Eizenga GC, Wright MH, Ali ML, Price AH, Norton GJ, Islam MR, Reynolds A, Mezey J (2011). Genome-wide association mapping reveals a rich genetic architecture of complex traits in Oryza sativa. Nat Commun..

[33] Wu D, Sato K, Ma JF (2015). Genome-wide association mapping of cadmium accumulation in different organs of barley. New Phytol..

[34] Zhao J, Yang W, Zhang S, Yang T, Liu Q, Dong J, Fu H, Mao X, Liu B (2018). Genome-wide association study and candidate gene analysis of rice cadmium accumulation in grain in a diverse rice collection. Rice..

[35] Zhang H, Zhang D, Wang M, Sun J, Qi Y, Li J, Wei X, Han L, Qiu Z, Tang S (2011). A core collection and mini core collection of Oryza sativa L. in China. Theor Appl Genet..

[36] Jianping Y, Jinli M, Zhanying Z, Haiyan X, Xiaoyang Z. Alternative splicing of OsLG3b controls grain length and yield in japonica rice. Plant Biotechnol J. 2018;16(9):1667-78. 10.1111/pbi.12903 PMC6097128 29479793

[37] Chen Z, Feng Y, Wang Y, Li Y, Liu Q, Xu S, Guan W (2015). Study on the growth and photosynthetic characteristics of wheat seedlings under [C_4_mim][OAc] (1-butyl-3-methyl-imidazolium acetate) with Cd^2+^ stress. B Environ Contamin Tox..

[38] Kollarova K, Kamenicka V, Vatehova Z, Liskova D (2018). Impact of galactoglucomannan oligosaccharides and Cd stress on maize root growth parameters, morphology, and structure. J Plant Physiol..

[39] Kang HM, Sul JH, Zaitlen NA, Kong SY, Freimer NB, Sabatti C, Eskin E, Service SK (2010). Variance component model to account for sample structure in genome-wide association studies. Nat Genet..

[40] Zhao FJ, Huang XY (2018). Cadmium Phytoremediation: Call Rice CAL1. Mol Plant..

[41] Miyadate H, Adachi S, Hiraizumi A, Tezuka K, Nakazawa N, Kawamoto T, Katou K, Kodama I, Sakurai K, Takahashi H (2011). OsHMA3, a P1B-type of ATPase affects root-to-shoot cadmium translocation in rice by mediating efflux into vacuoles. New Phytol..

[42] Chen J, Zou W, Meng L, Fan X, Ye G (2019). Advances in the uptake and transport mechanisms and QTLs mapping of cadmium in rice. Int J Mol Sci..

[43] Ueno D, Kono I, Yokosho K, Ando T, Yano M, Ma JF. A major quantitative trait locus controlling cadmium translocation in rice (Oryza sativa). New Phytol. 2009;182(3):644-53. 10.1111/j.1469-8137.2009.02784.x 19309445

[44] Wang J, Fang Y, Tian B, Zhang X, Zeng D, Guo L, Jiang H, Xue D (2018). New QTLs identified for leaf correlative traits in rice seedlings under cadmium stress. Plant Growth Regul..

[45] Zou W, Leng Y, Li J, Meng L, He H, Chen J, et al. Uptake and translocation mechanism of cadmium accumulation in rice and QTL mapping related to cadmium stress. Mol Plant Breed. 2018;16(24):8128-41.

[46] Xue D, Chen M, Zhang G (2009). Mapping of QTLs associated with cadmium tolerance and accumulation during seedling stage in rice (Oryza sativa L.). Euphytica.

[47] Huang Y, Sun C, Min J, Chen Y, Tong C, Bao J (2015). Association mapping of quantitative trait loci for mineral element contents in whole grain rice (Oryza *sativa* L.). J Agric Food Chem..

[48] Liu CL, Gao ZY, Shang LG, Yang CH, Ruan BP, Zeng DL, et al. Natural variation in the promoter of *OsHMA3* contributes to differential grain cadmium accumulation between Indica and Japonica rice. J Int Plant Biol. 2020;62(3):314-29. 10.1111/jipb.12794 30791211

[49] Bhatnagar A, Sillanp M (2009). Applications of chitin- and chitosan-derivatives for the detoxification of water and wastewater--a short review. Adv Colloid Interfac..

[50] Hirano S. Chitin biotechnology applications. Biotechnol Annu Rev. 1996;2:237-58. 10.1016/s1387-2656(08)70012-7 9704098

[51] Ngo DH, Kim SK. Chapter Two - Antioxidant Effects of Chitin, Chitosan, and Their Derivatives: Elsevier Sci Technol; 2014. 10.1016/B978-0-12-800268-1.00002-0 25300540

[52] Yong SK, Shrivastava M, Srivastava P, Kunhikrishnan A, Bolan N (2015). Environmental applications of chitosan and its derivatives. Rev Environ Contam T.

[53] Agati G, Azzarello E, Pollastri S, Tattini M (2012). Flavonoids as antioxidants in plants: location and functional significance. Plant Sci..

[54] Li L, Hou M, Cao L, Xia Y, Shen ZG, Hu Z (2018). Glutathione S-transferases modulate Cu tolerance in Oryza sativa. Environ Exp Bot..

[55] Zhang Y, Chen K, Zhao F-J, Sun C, Jin C, Shi Y, Sun Y, Li Y, Yang M, Jing X (2018). OsATX1 interacts with heavy metal P1B-type ATPases and affects copper transport and distribution. Plant Physiol..

[56] Hernández LE, Sobrinoplata J, Monteropalmero MB, Carrascogil S, Florescáceres ML, Ortegavillasante C, Escobar C (2015). Contribution of glutathione to the control of cellular redox homeostasis under toxic metal and metalloid stress. J Exp Bot..

[57] Li X, Guo Z, Lv Y, Cen X, Ding X, Wu H, Li X, Huang J, Xiong L (2017). Genetic control of the root system in rice under normal and drought stress conditions by genome-wide association study. PLoS Genet..

[58] Sasaki A, Yamaji N, Ma JF (2014). Overexpression of *OsHMA3* enhances Cd tolerance and expression of Zn transporter genes in rice. J Exp Bot..

[59] Lin SJ, Culotta VC (1995). The *ATX1* gene of Saccharomyces cerevisiae encodes a small metal homeostasis factor that protects cells against reactive oxygen toxicity. Proc Natl Acad of Sci U S A..

[60] Heo DH, Baek IJ, Kang HJ, Kim JH, Chang M, Kang CM, Yun CW (2012). Cd^2+^ binds to Atx1 and affects the physical interaction between Atx1 and Ccc2 in Saccharomyces cerevisiae. Biotechnol Lett..

[61] Shin LJ, Lo JC, Yeh KC (2012). Copper chaperone antioxidant protein1 is essential for copper homeostasis. Plant Physiol..

[62] Shin LJ, Yeh KC (2012). Overexpression of Arabidopsis *ATX1* retards plant growth under severe copper deficiency. Plant Signal Behav..

[63] Zhao FJ, Ma JF, Meharg AA, Mcgrath SP (2009). Arsenic uptake and metabolism in plants. New Phytol..

[64] Xu N, Chu Y, Chen H, Li X, Wu Q, Jin L, Wang G, Huang J (2018). Rice transcription factor OsMADS25 modulates root growth and confers salinity tolerance via the ABA–mediated regulatory pathway and ROS scavenging. PLoS Genet..

[65] Wu X, Chen J, Yue X, Wei X, Zou J, Chen Y, Su N, Cui J (2019). The zinc-regulated protein (ZIP) family genes and glutathione s-transferase (GST) family genes play roles in Cd resistance and accumulation of pak choi (Brassica campestris ssp. chinensis). Ecotox Environ Safe.

[66] Li JY, Wang J, Zeigler RS (2014). The 3,000 rice genomes project: new opportunities and challenges for future rice research. Gigascience.

[67] Li MX, Yeung JMY, Cherny SS, Sham PC, Li MX, Yeung JM, Cherny SS, Sham PC (2011). Evaluating the effective numbers of independent tests and significant p-value thresholds in commercial genotyping arrays and public imputation reference datasets. Hum Genet..

[68] Purcell S, Neale B, Todd-Brown K, Thomas L, Ferreira MA, Bender D, Maller J, Sklar P, de Bakker PI, Daly MJ (2007). PLINK: a tool set for whole-genome association and population-based linkage analyses. Am J Hum Genet..

[69] Trapnell C, RA, Goff L, Pertea G, Kim D, Kelley DR (2012). Differential gene and transcript expression analysis of RNA-seq experiments with TopHat and Cufflinks. Nat Protoc..

[70] Kawahara Y, Bastide MDL, Hamilton JP, Kanamori H (2013). Improvement of the Oryza *sativa* Nipponbare reference genome using next generation sequence and optical map data. Rice.

[71] Benjamini Y, Dan D, Elmer G, Kafkafi N, Golani I (2001). Controlling the false discovery rate in behavior genetics research. Behav Brain Res..

[72] Tian T, Liu Y, Yan H, You Q, Yi X, Du Z, Xu W, Su Z (2017). agriGO v2.0: a GO analysis toolkit for the agricultural community, 2017 update. Nucleic Acids Res..

[73] Wang X, Ning Y, Zhang P, Li C, Zhou R, Guo X. Hair multi-bioelement profile of Kashin-Beck disease in the endemic regions of China. J Trace Elem Med Biol. 2019;54:79-97. 10.1016/j.jtemb.2019.04.002 31109624

[74] Zhao Y, Zhang H, Xu J, Jiang C, Yin Z, Xiong H, Xie J, Wang X, Zhu X, Li Y (2018). Loci and natural alleles underlying robust roots and adaptive domestication of upland ecotype rice in aerobic conditions. PLoS Genet..

