# Supplementary figures and images for: Loci and natural alleles for cadmium-mediated growth responses revealed by a genome wide association study and transcriptome analysis in rice
Source: BMC Plant Biol. 2021 Aug 13;21:374. doi: 10.1186/s12870-021-03145-9 (PMC8362254; doi:10.1186/s12870-021-03145-9)

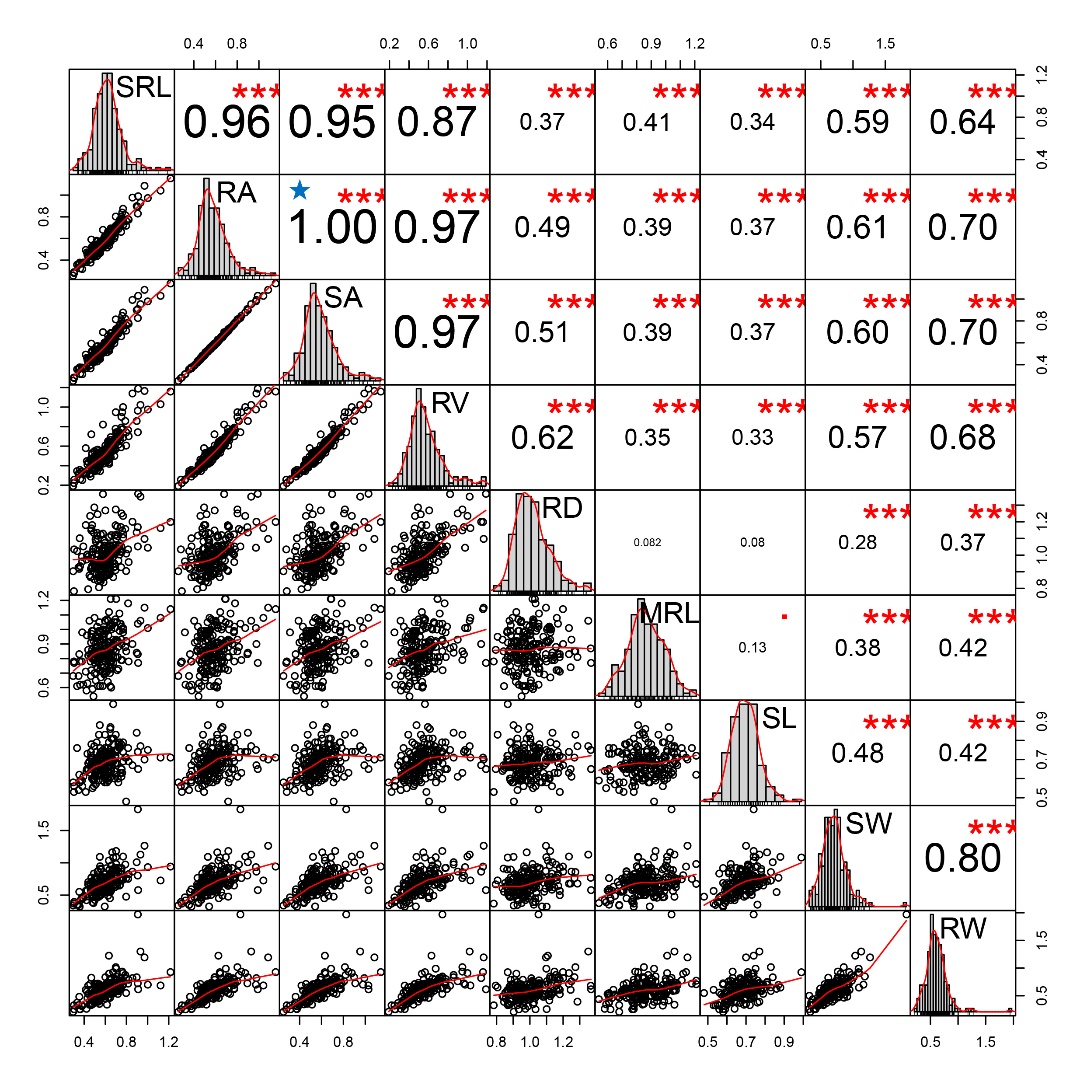

Supplement: Supplementary file 1 — Additional file 1: Fig. S1. Correlation coefficient among 9 traits related to high cadmium-mediated growth responses. [file 12870_2021_3145_MOESM1_ESM.docx]

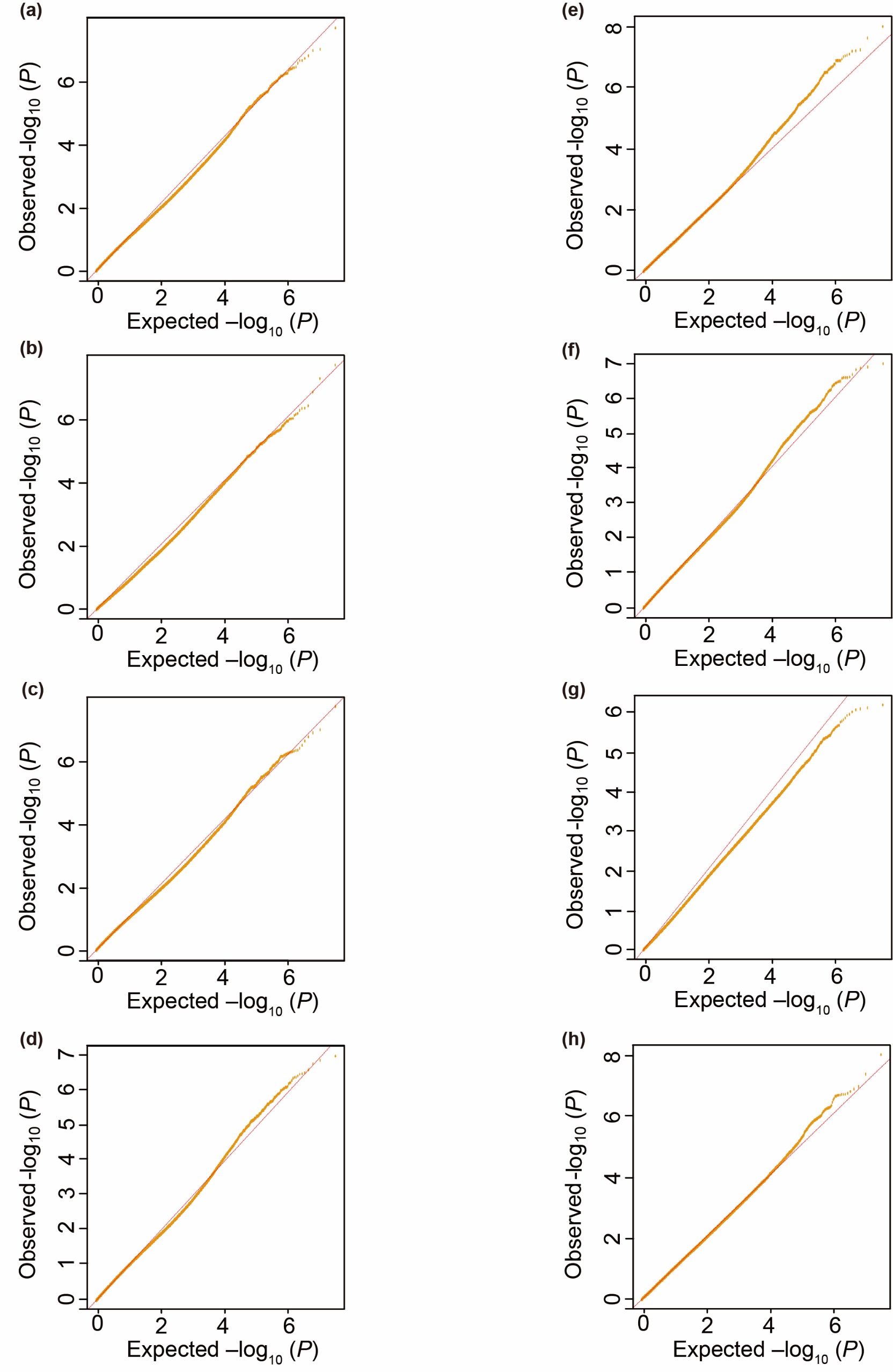

Supplement: Supplementary file 2 — Additional file 2: Fig S2. Quantile-quantile plot for SA (a), SRL (b), RA (c), RV (d), SW (e), RD (f), MRL (g), and SL (h). [file 12870_2021_3145_MOESM2_ESM.docx]

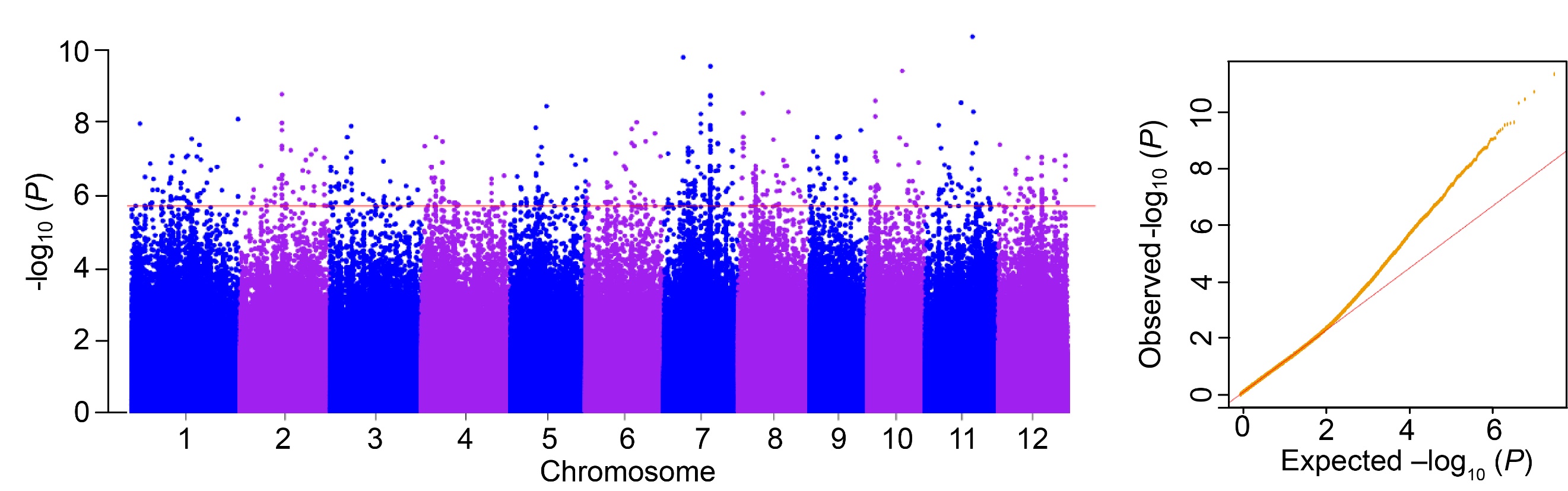

Supplement: Supplementary file 3 — Additional file 3: Fig. S3. Identification of RW QTLs for cadmium-mediated growth responses by GWAS. [file 12870_2021_3145_MOESM3_ESM.docx]

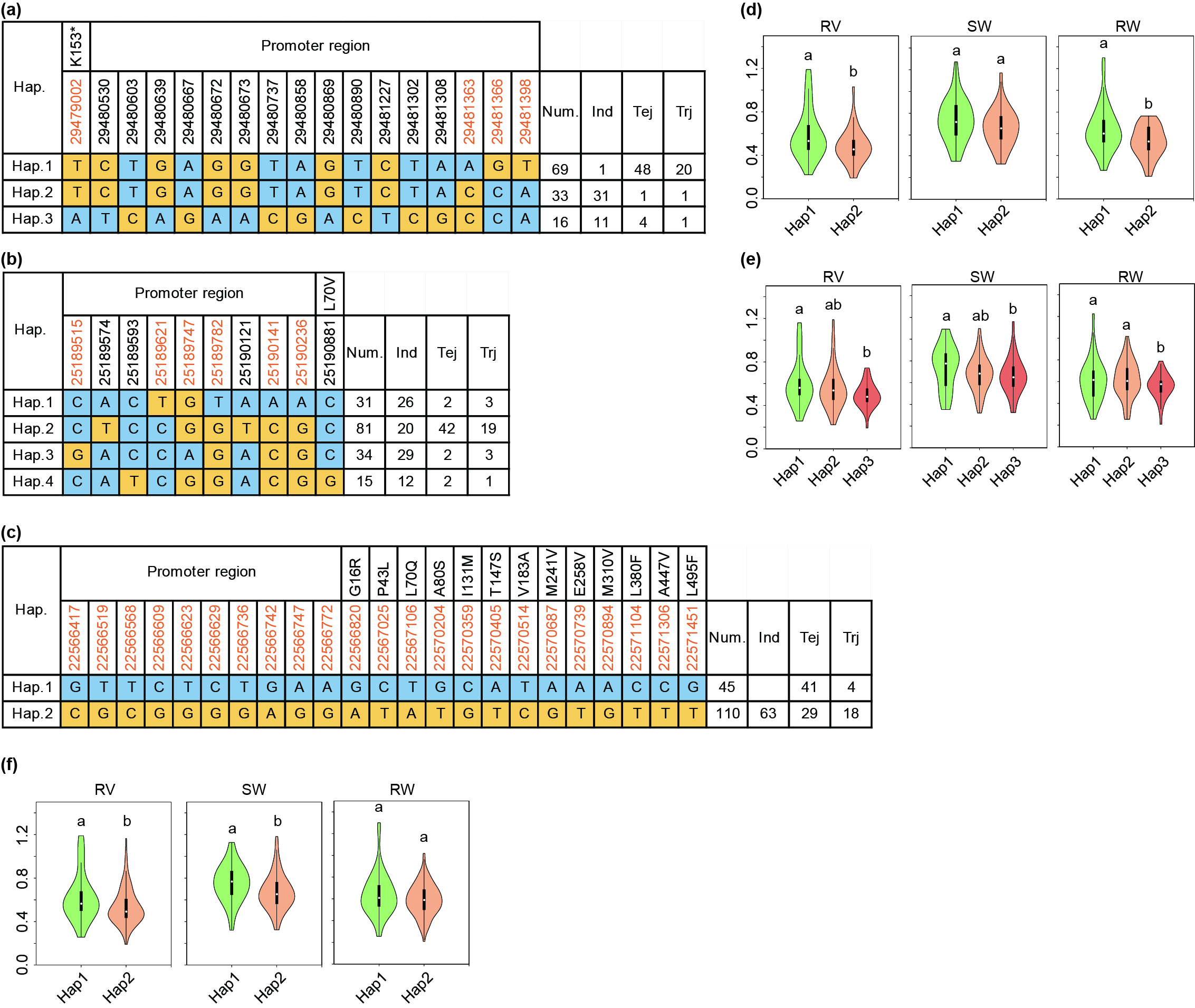

Supplement: Supplementary file 4 — Additional file 4: Fig. S4. Haplotype analysis of OsHMA2, CAL1 and OsLCT1. [file 12870_2021_3145_MOESM4_ESM.docx]

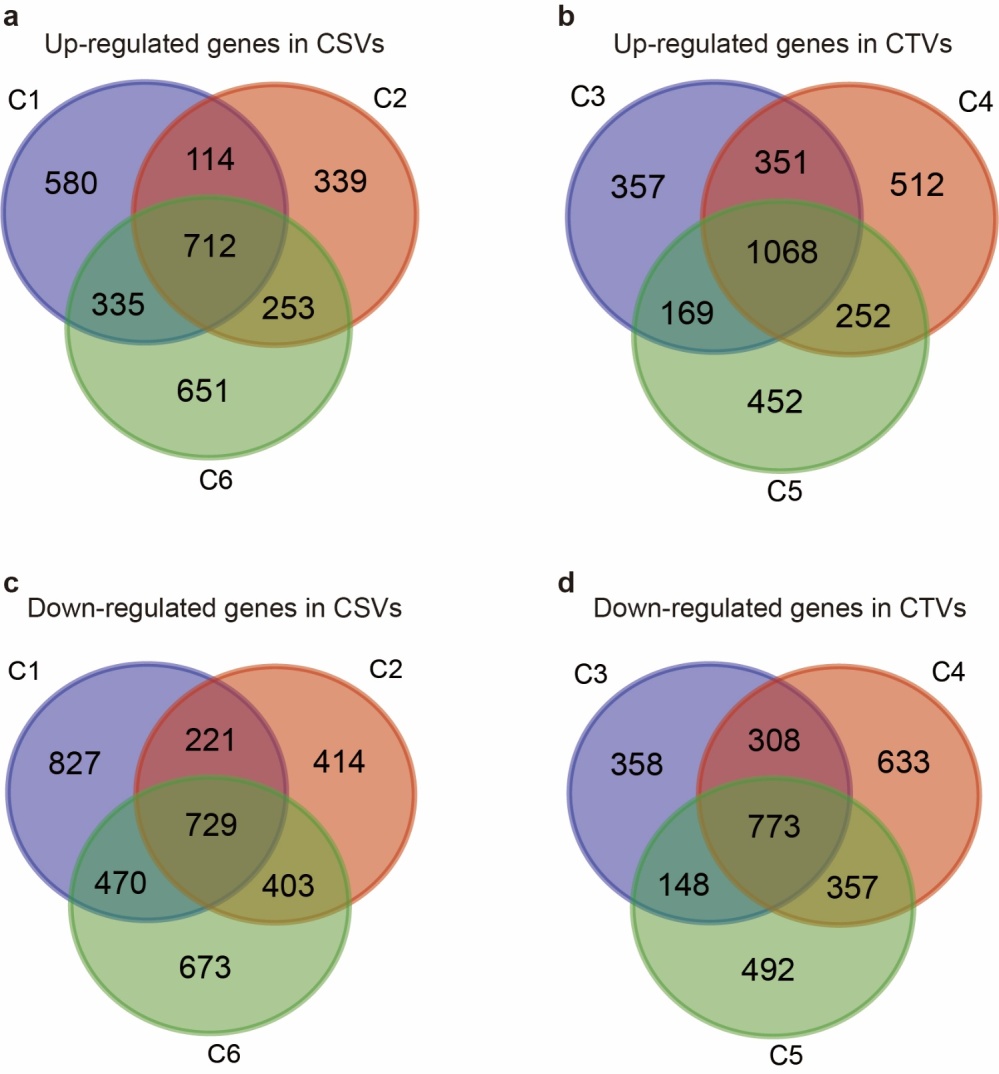

Supplement: Supplementary file 5 — Additional file 5: Fig. S5. Modified Venn diagrams showing the common and specific Cd-responsive genes across different Cd-tolerant and Cd-sensitive rice varieties. [file 12870_2021_3145_MOESM5_ESM.docx]

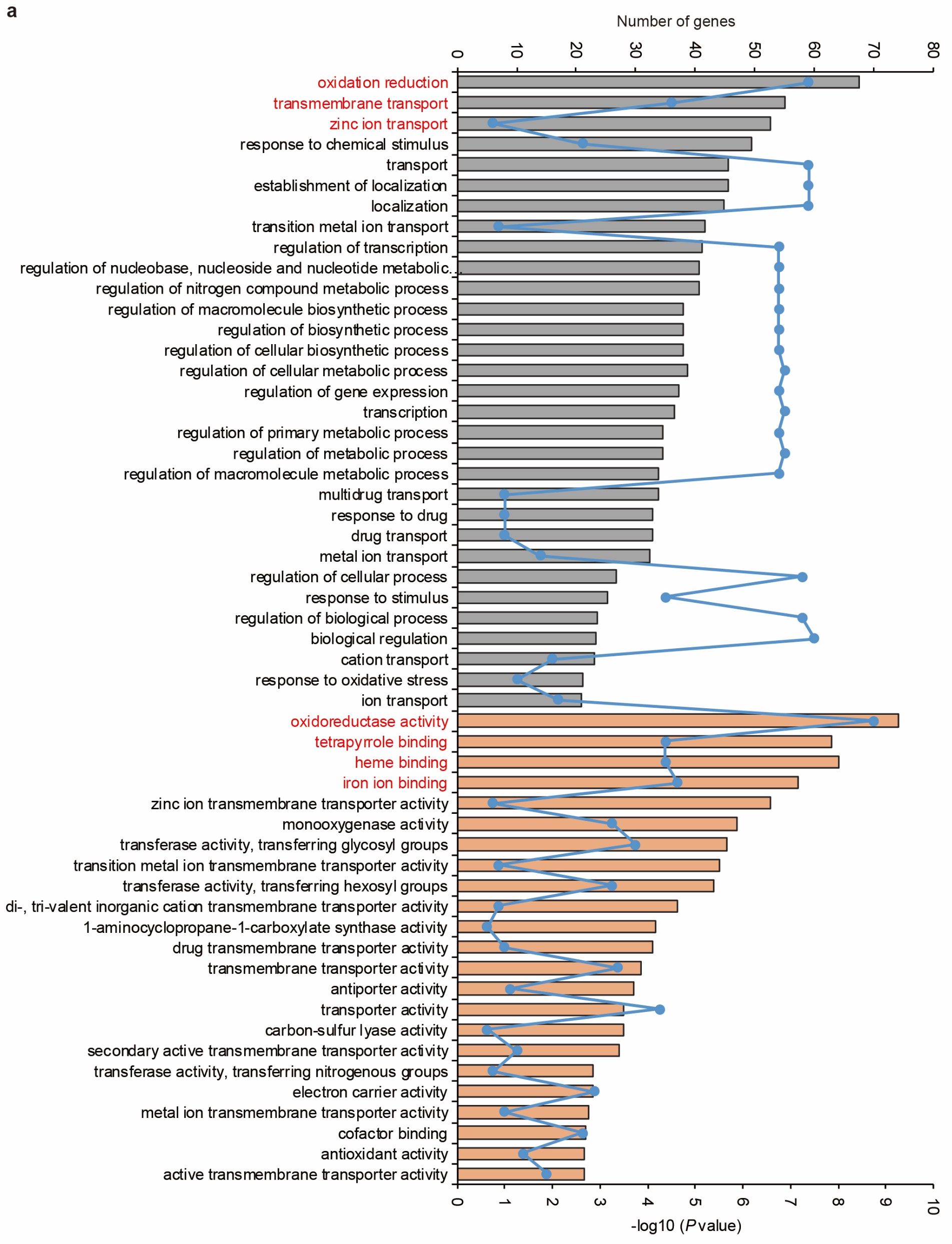


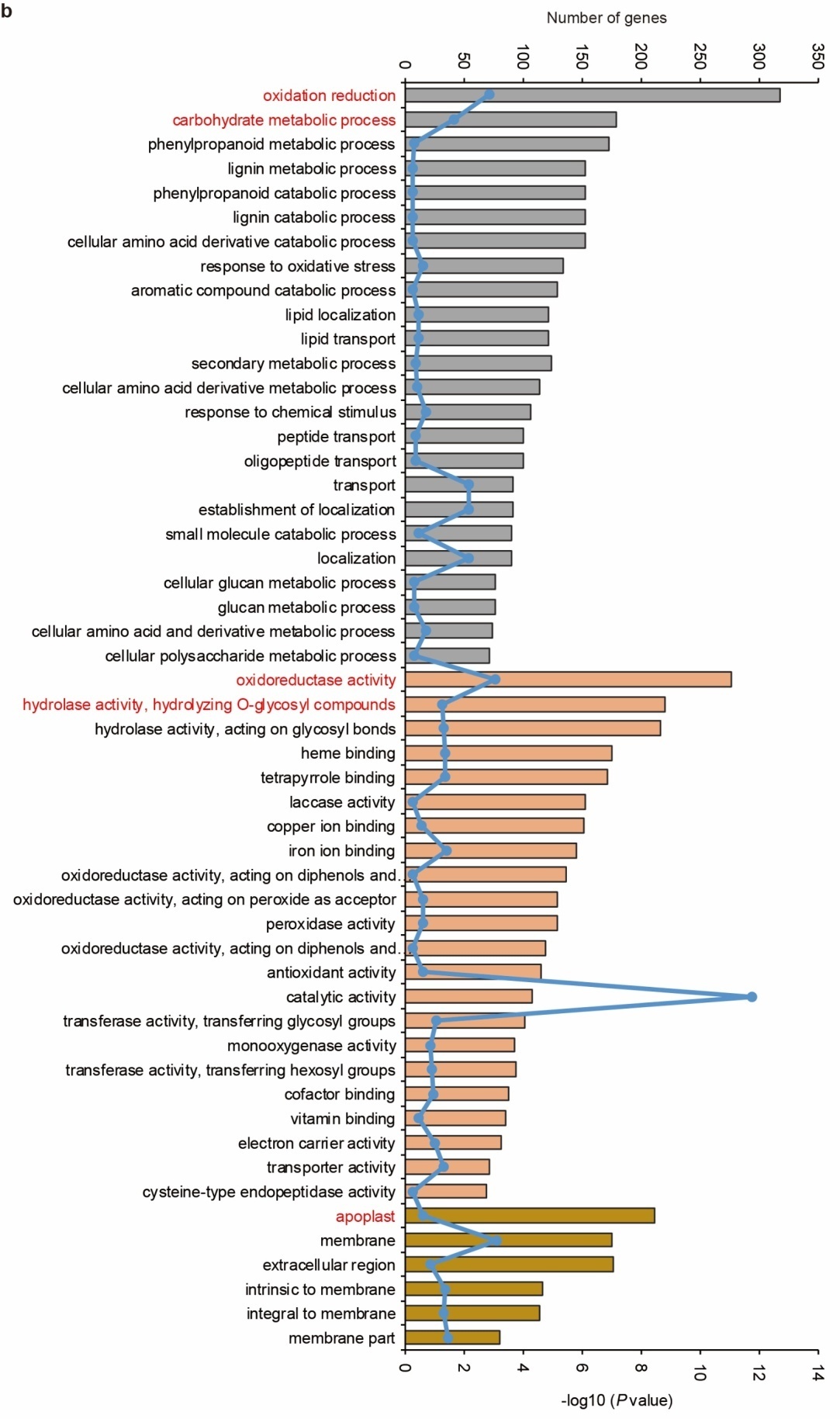

Supplement: Supplementary file 6 — Additional file 6: Fig. S6. GO enrichment analysis of DEGs in response to Cd stress in CSVs. [file 12870_2021_3145_MOESM6_ESM.docx]

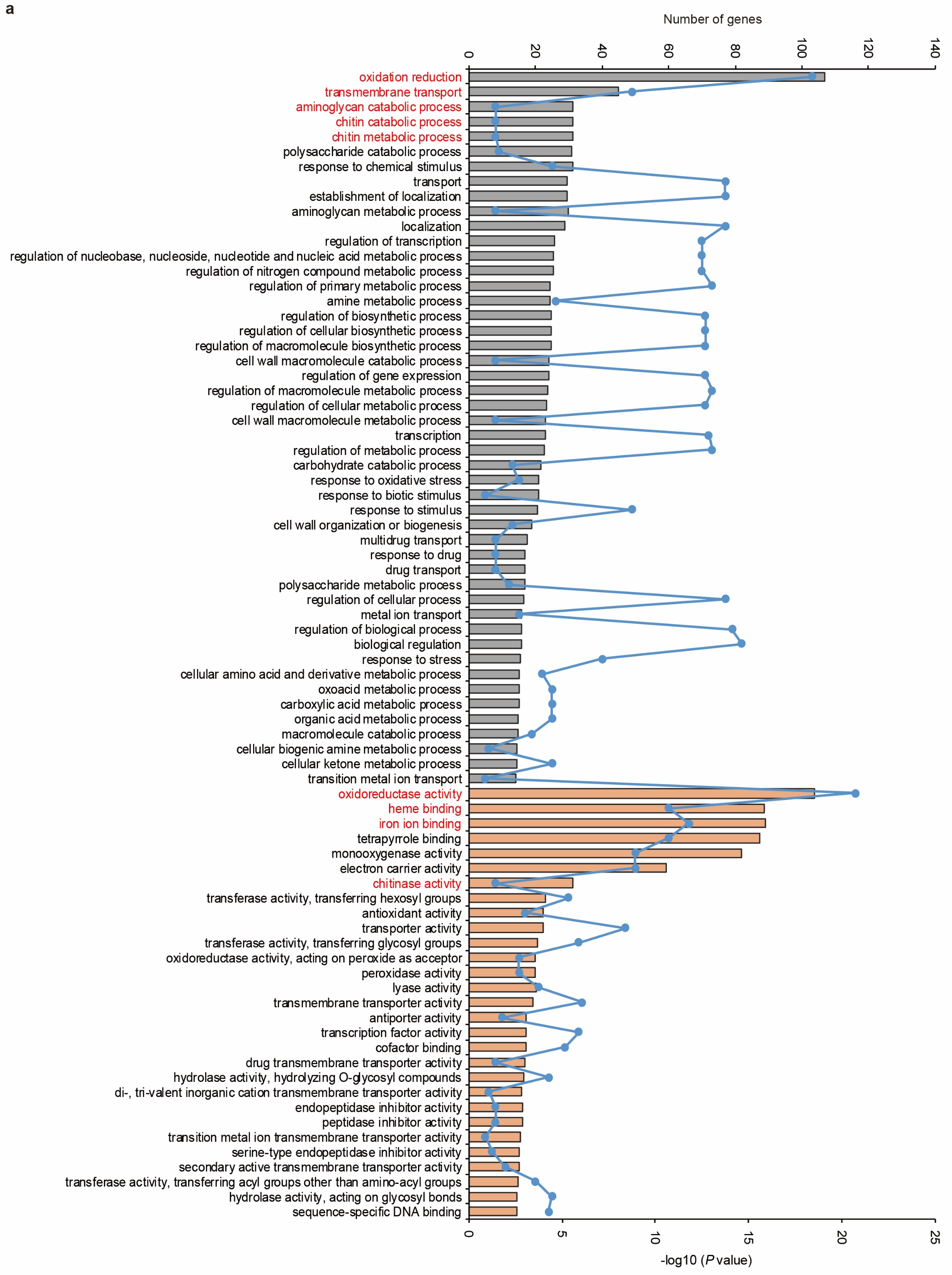


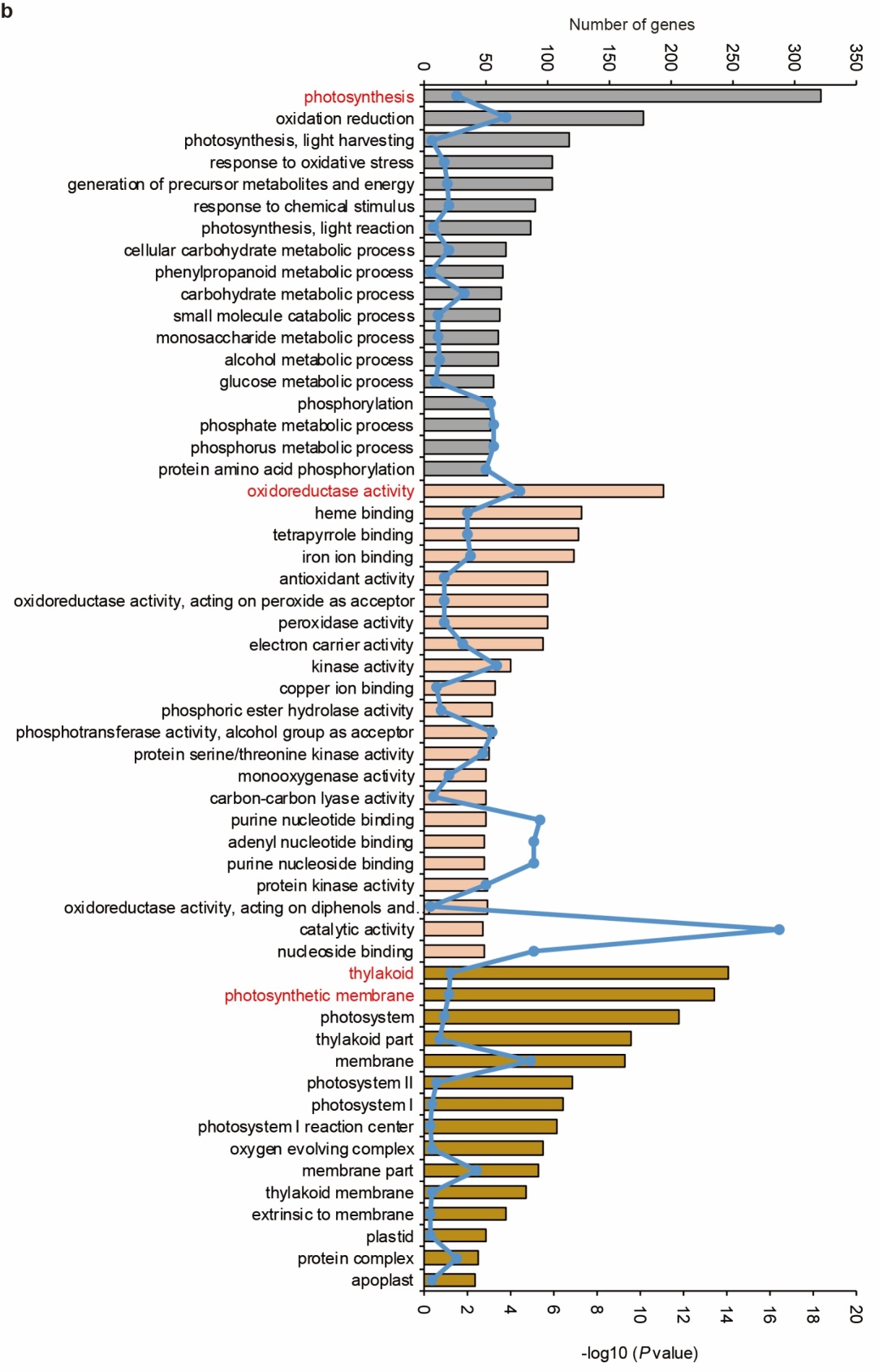

Supplement: Supplementary file 7 — Additional file 7: Fig. S7. GO enrichment analysis of DEGs in response to Cd stress in CTVs. [file 12870_2021_3145_MOESM7_ESM.docx]

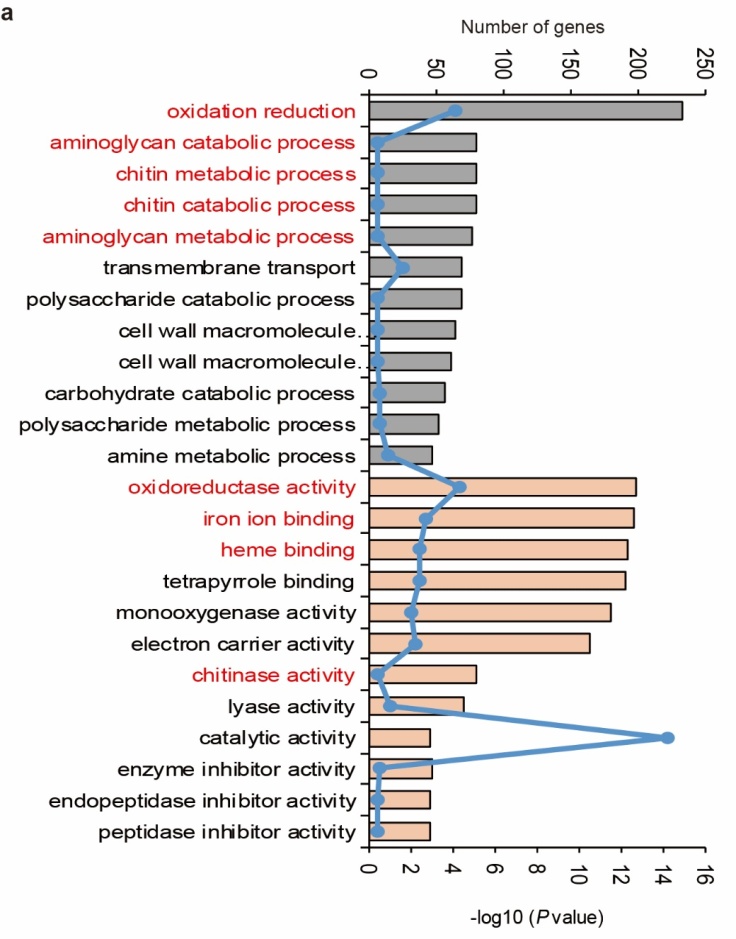


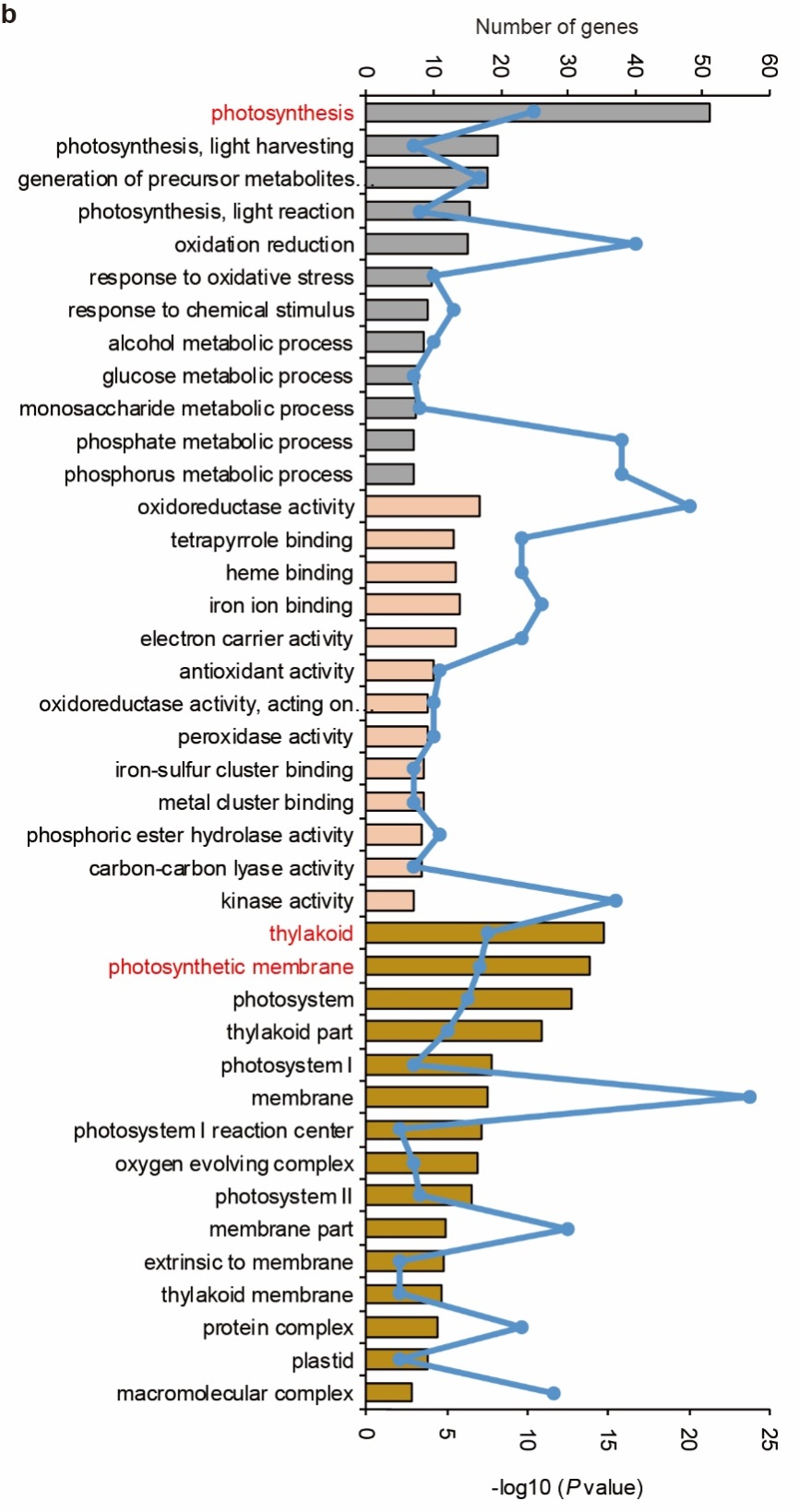

Supplement: Supplementary file 8 — Additional file 8: Fig. S8. GO enrichment analysis of specific Cd-responsive genes in CTVs. [file 12870_2021_3145_MOESM8_ESM.docx]

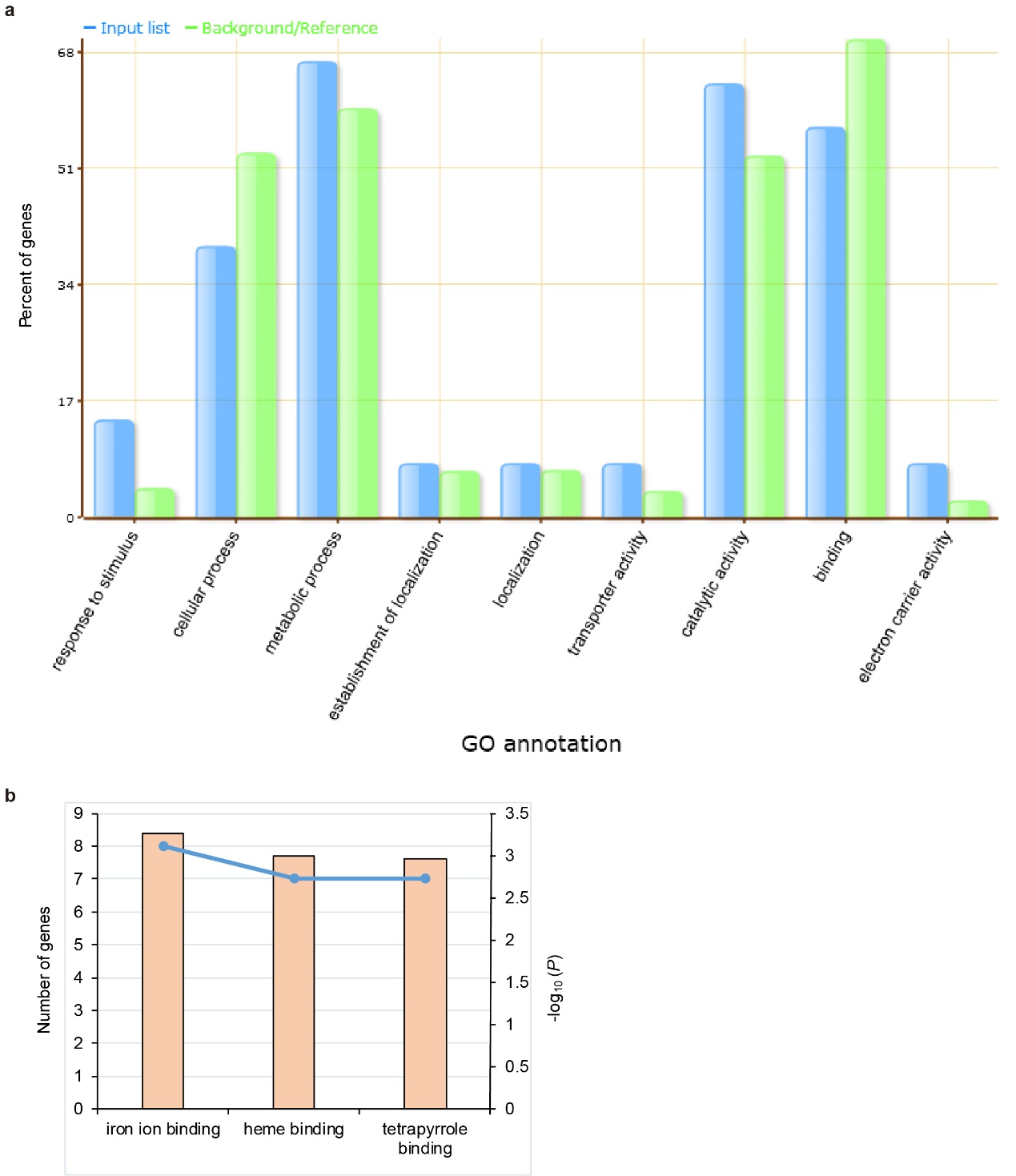

Supplement: Supplementary file 9 — Additional file 9: Fig. S9. GO enrichment analysis of common genes between GWAS and RNA-seq. [file 12870_2021_3145_MOESM9_ESM.docx]

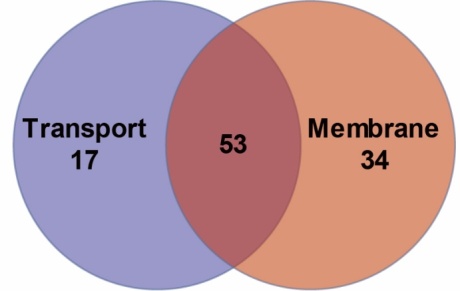

Supplement: Supplementary file 10 — Additional file 10: Fig. S10. The numbers of genes annotated with transport and membrane from the Go Slim assignments for annotated genes. [file 12870_2021_3145_MOESM10_ESM.docx]

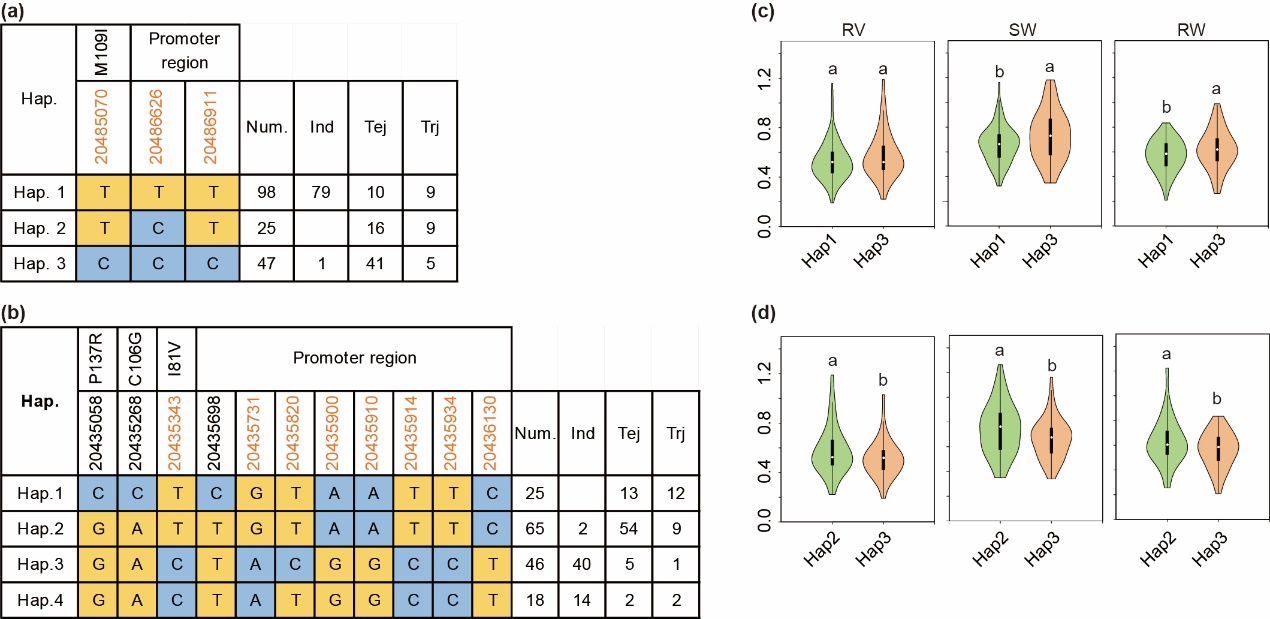

Supplement: Supplementary file 11 — Additional file 11: Fig. S11. Haplotype analyses of two QTL genes for cadmium-mediated growth responses. [file 12870_2021_3145_MOESM11_ESM.docx]

**
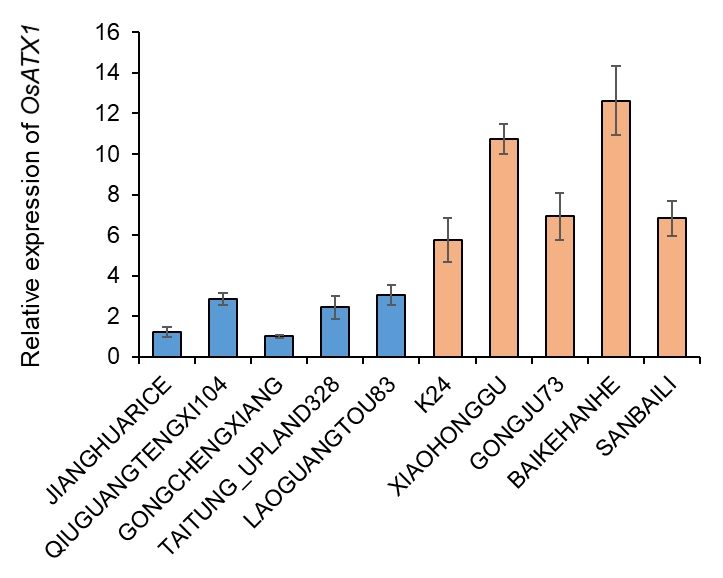
**

Supplement: Supplementary file 12 — Additional file 12: Fig. S12. Expression analyses of OsATX1 in natural rice varieties. [file 12870_2021_3145_MOESM12_ESM.docx]
